# The genome of *Leishmania adleri* from a mammalian host highlights chromosome fission in *Sauroleishmania*

**DOI:** 10.1038/srep43747

**Published:** 2017-03-03

**Authors:** Simone Coughlan, Peter Mulhair, Mandy Sanders, Gabriele Schonian, James A. Cotton, Tim Downing

**Affiliations:** 1 School of Mathematics, Applied Mathematics and Statistics, National University of Ireland, Galway, Ireland; 2 School of Biotechnology, Dublin City University, Dublin, Ireland; 3 Wellcome Trust Sanger Institute, Hinxton, UK; 4 Charite University Medicine, Berlin, Germany

## Abstract

Control of pathogens arising from humans, livestock and wild animals can be enhanced by genome-based investigation. Phylogenetically classifying and optimal construction of these genomes using short sequence reads are key to this process. We examined the mammal-infecting unicellular parasite *Leishmania adleri* belonging to the lizard-infecting *Sauroleishmania* subgenus. *L. adleri* has been associated with cutaneous disease in humans, but can be asymptomatic in wild animals. We sequenced, assembled and investigated the *L. adleri* genome isolated from an asymptomatic Ethiopian rodent (MARV/ET/75/HO174) and verified it as *L. adleri* by comparison with other *Sauroleishmania* species. Chromosome-level scaffolding was achieved by combining reference-guided with *de novo* assembly followed by extensive improvement steps to produce a final draft genome with contiguity comparable with other references. *L. tarentolae* and *L. major* genome annotation was transferred and these gene models were manually verified and improved. This first high-quality draft *Leishmania adleri* reference genome is also the first *Sauroleishmania* genome from a non-reptilian host. Comparison of the *L. adleri* HO174 genome with those of *L. tarentolae* Parrot-TarII and lizard-infecting *L. adleri* RLAT/KE/1957/SKINK-7 showed extensive gene amplifications, pervasive aneuploidy, and fission of chromosomes 30 and 36. There was little genetic differentiation between *L. adleri* extracted from mammals and reptiles, highlighting challenges for leishmaniasis surveillance.

*Leishmania* are the protozoan parasites responsible for causing the neglected tropical disease leishmaniasis that affects 1.2–2.0 million people annually (WHO 2016), and they also infect livestock, pets and wild animals^1^. These trypanosomatids typically have a digenetic life cycle existing as flagellated promastigotes in the sandfly vector and as intracellular amastigotes in vertebrate hosts or, possibly, as promastigotes in reptiles^2^. Over 20 *Leishmania* species cause leishmaniasis, which presents diverse clinical pathologies: principally visceral leishmaniasis (VL), cutaneous leishmaniasis (CL) and mucocutaneous leishmaniasis^1^,^3^. VL causes ~50,000 deaths and infects 0.2–0.5 million annually. Localised, diffuse and disseminated CL affects 0.7–1.2 million/year, though this is probably underestimated^4^. Most infected hosts are asymptomatic carriers that can transmit the parasite to sandflies, and must be targeted to eliminate leishmaniasis.

The genus *Leishmania* contains 53 known species, initially classified according to the sandfly gut region inhabited by the parasites^5^ where those developing in the fore-and mid-gut are one subgenus (*Leishmania*). Parasites developing in the hind-gut form four groups: three are subgenera *Paraleishmania, Sauroleishmania* and *Viannia*, and the fourth set is the *L. enriettii* species complex. The origin of these groups may be at the breakup of the Gondwana supercontinent^6^, and the most basal one (*Paraleishmania*) is only in South America. Most genus *Leishmania* parasites infect insect vectors and mammal hosts, but *Sauroleishmania* are an exception because their vertebrate hosts are primarily lizards. This host range is particularly striking because *Sauroleishmania* are closely related to the *Leishmania* subgenus^7^, with whom they share a common ancestor ~42 million years ago (95% CI 24–65 mya). Extant *Sauroleishmania* began diversifying ~16 mya^6^ into 19 named and two unnamed species found in Asia and Africa^8^.

*Sauroleishmania* are globally distributed and a wide range of sandflies and animals act as vectors and hosts, respectively^9^. *L. adleri* is associated with lizard- and mammal-feeding vector *Phlebotomus clydei* in Kenya^10^ and *Sergentomyia (Sergentomyia) dentata* in Iran^11^. Diverse *Sauroleishmania* infections were described for different lacertid, agamid and gecko desert lizard species, including *L. gymnodactyli* in North-western China^12^. *Sauroleishmania* were isolated from vectors^11^ and lizards^13^ in Iran, and *L. tarentolae* were found in reptile-biting *S. (S.) minuta* sandflies in Spain^14^.

The phylogenetic position of *Sauroleishmania* between the mammal-infecting subgenera *Leishmania* and *Viannia*^15^,^16^,^17^ suggests that it represents a widespread lineage of *Leishmania* that switched from mammals to reptiles as their main hosts. *Sauroleishmania* member *L. tarentolae* is a non-pathogenic lab model because it rapidly replicates in lizards. Another *Sauroleishmania* species, *L. adleri* undergoes development in the sandfly anterior mid-gut, but can cause transient CL in humans^18^, and cryptic infections as long as five weeks in hamsters and mice^19^. Similarly, *L. tarentolae* can invade human macrophages and may exist as amastigotes in mammals, though with slower replication^20^,^21^. *L. adleri* was the most likely cause of two CL cases in humans^22^, and human and canine *Sauroleishmania* VL infections from 1984–90 were found in China^23^. Amastigotes causing VL rather than CL may have been present in bone marrow and intestinal tissue samples from a 300-year-old mummy from Brazil based on a kinetoplastid DNA (kDNA) amplicon matching *L. tarentolae* Parrot-TarII but not other *Leishmania*^24^.

Leishmaniasis is the most common neglected tropical disease in East Africa, where tropical and sub-tropical climates sustain sandfly populations^25^. Areas with endemic leishmaniasis show that one in eight people have undergone VL treatment in the Eastern Gedaref state of Sudan^26^, and parts of Ethiopia in 2014 had a VL rate of 6.7 and a CL one of 0.8 per 1,000^27^. CL in this region is transmitted by *P. papatasi* or *dubosqui* sandflies^28^ and is frequently associated with *L. major*^28^. VL is transmitted by *P. orientalis* and is typically caused by *L. donovani* complex species^29^, though other *Leishmania* are implicated^8^.

Strain MARV/ET/1975/HO174 was originally isolated in the rural Setit Humera district of north-western Ethiopia, where *Acacia* and *Balanites* forests associated with *P. orientalis* are used as shelter for overnight sleeping^29^. The HO174 genome presented here was previously classified as an unusual *Leishmania* lineage based on multi-locus microsatellite typing (MLMT)^30^. HO174 was a parasite isolate from an asymptomatic Nile or African grass rat (*Arvicanthis niloticus*^22^, which is a reservoir for several *Leishmania* species and promotes the transmission of *L. donovani*^31^ and *L. major*^32^ in East Africa.

Genome assemblies have been published for many *Leishmania* species and isolates since the first *Leishmania* reference genome sequence of *L. major* MHOM/IL/1981/Friedlin^33^. These include *L. braziliensis* MHOM/BR/1975/M2904, *L. infantum* MCAN/ES/1998/LLM-87 (JPCM5)^34^, *L. donovani* MHOM/NP/2003/BPK282/0cl4^35^, *L. mexicana* MHOM/GT/2001/U1103cl25^36^, *L. amazonensis* MHOM/BR/1973/M2269^37^, and *L. panamensis* MHOM/PA/94/PSC-1^38^. The *L. major* genome is 32.8 Mb and contains 8,311 protein-coding genes^33^, and gene content, genome size and structure are largely conserved across *Leishmania*. Like other kinetoplastids, *Leishmania* genomes are comprised of polycistronic transcriptional units (PTUs) separated by strand-switch regions (SSRs) from which RNA polymerase II transcribes in both directions^39^. PTU expression is regulated by variable RNA stability through mRNA 5′-trans-splicing of the 39-nucleotide mini-exon splice-leader RNA and 3′-polyadenylation prior to translation^40^. This post-transcriptional regulation means that gene detection, context and copy number can provide insights into function.

The sole sequenced *Sauroleishmania* genome is for *L. tarentolae* RTAR/DZ/1939/Parrot-TarII isolated from the lizard *Tarentola mauritanica*^41^. It has 36 chromosomes, is aneuploid, and contains 8,530 genes. Its gene-level orthology and PTU arrangement are conserved with *L. major*^41^. Here, we generated whole-genome sequence data for MARV/ET/1975/HO174 that we assign as *L. adleri* (or a closely related species) using a combination of *de novo* and reference-guided assembly to create an annotated-directed improved draft genome^42^. We used this data and that of another *L. adleri* isolate (RLAT/KE/1957/SKINK-7) to characterise the evolution of *Sauroleishmania*, including genome rearrangements, chromosome structure and gene copy number.

## Results

### The annotated reference genome of HO174

Preliminary analysis suggested that MARV/ET/1975/HO174 was a member of the *Sauroleishmania* subgenus, based on mapping 18,183,113 paired-end Illumina HiSeq sequence 75 bp reads to existing reference genomes. Accordingly, we chose to generate a reference genome for this mammal-infecting *Sauroleishmania* to explore the genetic context of host specificity. The HO174 draft genome assembled using these reads has 69-fold median coverage and spans 30.35 Mb. Compared to the most closely related genome of *L. tarentolae* Parrot-TarII, it had fewer gaps (Table 1), fewer genes on chromosomes (7,570), more genes on unassigned contigs (389), and shorter chromosome lengths with the exceptions of chromosomes 4, 8, 9, 15, 21, 24 and 28 (Supplementary Fig. S1).

### Progressive improvement of the original HO174 assembly

After eliminating low-quality reads and contaminant screening, 17,644,995 read pairs were assembled into 18,480 contigs with an initial N50 of 4.7 Kb Velvet v1.2.09^43^. Iterative joining of these contigs by SSPACE v2.0^44^ resulted in 5,259 scaffolds with a N50 of 54.2 Kb (Supplementary Table S1). 4,834 (55%) of 8,786 initial gaps were closed^45^, and nucleotide errors were corrected^46^ (Supplementary Fig. S2). 627 false scaffold joins identified with REAPR^47^ increased the number of scaffolds to 5,785 with a N50 of 38.8 Kb and 89.1% error-free bases (EFBs). This EFB rate was similar to the reference genomes for *Caenorhabditis elegans* WS228 (90.3%), *Plasmodium falciparum* v3 (94.9%) and *Mus musculus* GRCm38 (80.1%). The scaffolds were contiguated into (initially) 36 pseudo-chromosomes using the *L. tarentolae* genome with ABACAS^48^. 250 contigs >1 Kb long and not assigned to chromosomes spanned 1.66 Mb. Fourteen contigs had homology to minicircle kDNA (290,165 bp), one to maxicircle kDNA (1,075 bp) and one to both minicircle and maxicircle kDNA (1,078 bp).

### HO174 represents *L. adleri* in the *Sauroleishmania* subgenus

**Figure 1:**
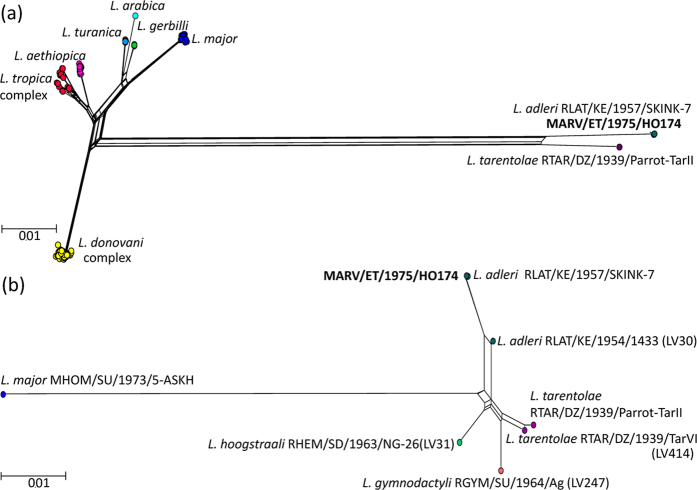
*L. adleri* HO174 was a *Sauroleishmania* isolate based on neighbornet network of the uncorrected p-distances of an alignment of: (**a**) seven concatenated genes with 4,677 sites for 225 strains. The scale bar indicates the number of substitutions per site. The *L. adleri* RLAT/KE/1957/SKINK-7 and MARV/ET/1975/HO174 nodes partially obscure each other. Compared to HO174, there are only two substitutions with SKINK-7, 177 substitutions with *L. tarentolae* RTAR/DZ/1939/Parrot-TarII, 635 with *L. major* MRHO/SU/1959/P-STRAIN, 627 with *L. infantum* MHOM/IT/1985/ISS175, 630 with *L. donovani* MHOM/YE/1993/LEM2677, and 599 substitutions with *L. tropica* MHOM/JO/1996/JH-88; (**b**) two concatenated genes (encoding DNA polymerase *α* catalytic polypeptide and RNA polymerase II largest subunit) with 2,192 sites of six samples. The scale bar indicates the number of substitutions per site. The SKINK-7 and HO174 nodes partially obscure each other. Compared to HO174, there are two substitutions with SKINK-7, 21 with *L. adleri* RLIZ/KE/1954/1433, 49 with *L. tarentolae* RTAR/DZ/1939/TarVI (from a *Tarentola* wall gecko) and *L. tarentolae* Parrot-TarII, 51 with *L. hoogstraali* RHEM/SD/1963/NG-26 (LV31), 55 with *L. gymnodactyli* RGYM/SU/1964/Ag (LV247) and 203 with *L. major* MHOM/SU/1973/5-ASKH. *L. adleri* SKINK-7 had the same number of substitutions as HO174 with each of these isolates.

The genus and species of the HO174 genome was assessed using sequences for seven genes (4,677 sites) from 222 isolates from infected patients, mammals and insect vectors for ten *Leishmania* species^49^. Orthologous genes were extracted using BLASTn for the HO174 genome, *L. tarentolae* genome^41^ and a *L. adleri* RLAT/KE/1957/SKINK-7 assembly created using Velvet^43^: SKINK-7 was originally from a long-tailed lizard (*Latastia longicaudata*), injected into hamsters, and isolated from a rodent spleen. The orthologs were aligned with the 222^50^ to construct a network^51^. HO174 was most closely related to SKINK-7 with just two substitutions, compared with 177 between HO174 and *L. tarentolae*, and 177 between SKINK-7 and *L. tarentolae* (Fig. 1a).

**Figure 2:**
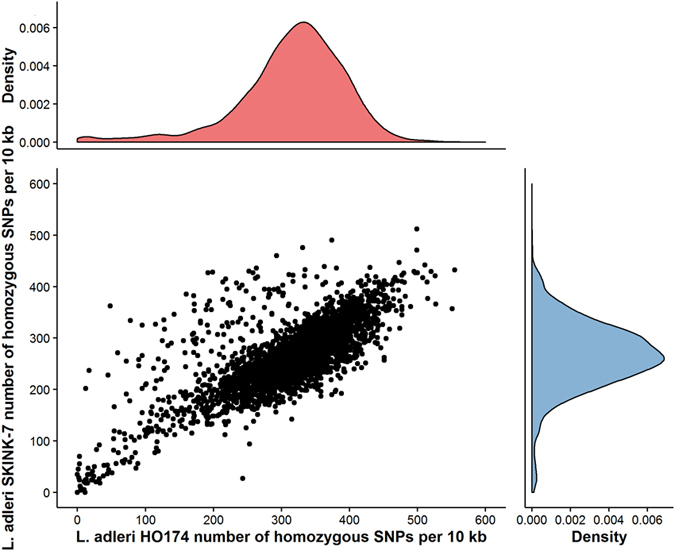
Divergence of *L. adleri* HO174 and *L. adleri* SKINK-7 from *L. tarentolae* as the number of homozygous SNPs per 10 Kb window. Loci with high divergence in both genomes are at the top right. Density plots of divergence per 10 Kb of HO174 and SKINK-7 indicated that HO174 was more divergent from *L. tarentolae* than SKINK-7.

We propose that HO174 is a mammalian isolate of *L. adleri* on the basis of its phylogenetic placement within *Sauroleishmania*. Two genes previously sequenced, DNA polymerase α catalytic polypeptide (LaHO174_161460) and RNA polymerase II largest subunit (LaHO174_310170)^7^, were aligned for HO174, *L. adleri* SKINK-7, *L. tarentolae* Parrot-TarII, *L. tarentolae* RTAR/DZ/1939/LV414, *L. adleri* RLIZ/KE/1954/1433 (LV30) isolated from a *Latastia* lizard, *L. hoogstraali* RHEM/SD/1963/NG-26 (LV31) from a Mediterranean house gecko (*Hemidactylus turcicus*), and *L. gymnodactyli* RGYM/SU/1964/Ag (LV247) from agamid lizard promastigotes (*Agama sanguinolenta,* aka *Trapelus sanguinolentus*) in Turkmenistan that was not pathogenic in mammals^52^. HO174 was most closely related to *L. adleri* SKINK-7 with two substitutions and *L. adleri* 1433 with 21 substitutions (Fig. 1b), whereas the others were more divergent (49 for both *L. tarentolae*, 51 for *L. hoogstraali* NG-26, 55 for *L. gymnodactyli* Ag, Supplementary Table S2). Genome-wide SNP rates confirmed the older common ancestry of HO174 with *L. tarentolae* (999,834 SNPs, 35.8 SNPs/Kb) and SKINK-7 (855,686 SNPs, 30.6 SNPs/Kb) (Fig. 2a) compared to that of HO174 with SKINK-7 (36,254 or 1.3 SNPs/Kb - SKINK7 had 15,816 heterozygous SNPs). Similarly, SKINK-7 showed less divergence to *L. tarentolae* than HO174 (Fig. 2b).

### Two ancestral *L. adleri* chromosome fission events produce 38 chromosomes

**Figure 3:**
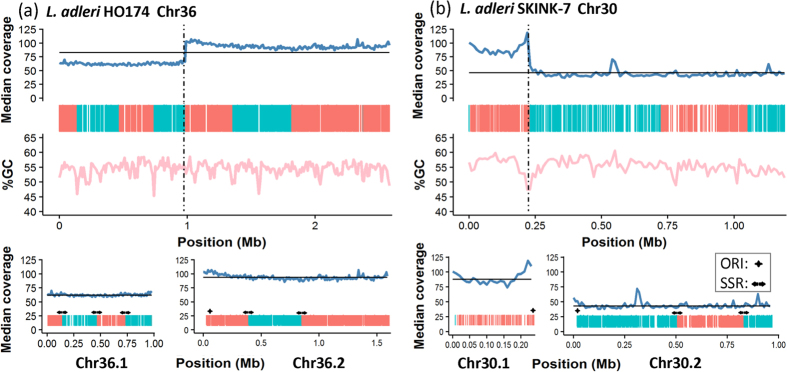
Evidence of chromosome fission of (**a**) *L. adleri* HO174 chromosome 36 into 36.1 and 36.2; and (**b**) *L. adleri* SKINK-7 chromosome 30 into 30.1 and 30.2. Median read coverage (blue) and GC content (pink) were measured in 10 Kb blocks. Black horizontal lines indicate median coverage of that chromosome. The dashed line indicates the fission breakpoints on the original chromosomes: at 989,697 for chromosome 36 and 230,911 for chromosome 30. Genes transcribed from left to right (green) and from right to left (red) are homologous to *L. major* polycistronic transcriptional units from Thomas *et al*.^67^ with their transcription SSRs shown as arrows and origins of replication shown as black crosses.

**Figure 4:**
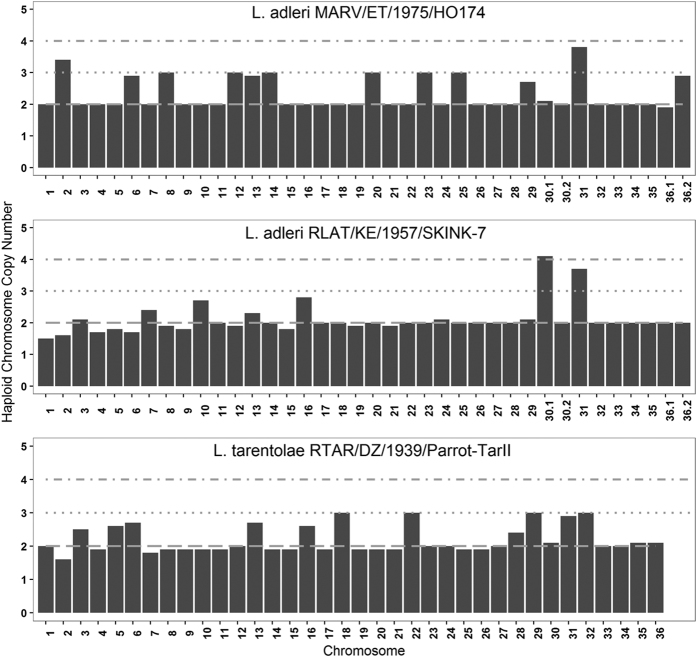
Chromosome copy numbers based on haploid median read coverage for *L.* *adleri* HO174 reads mapped to the *L. adleri* HO174 reference (top); *L. adleri* SKINK-7 reads mapped to the *L. adleri* HO174 reference (middle); and *L. tarentolae* mapped to itself (bottom).

**Figure 5:**
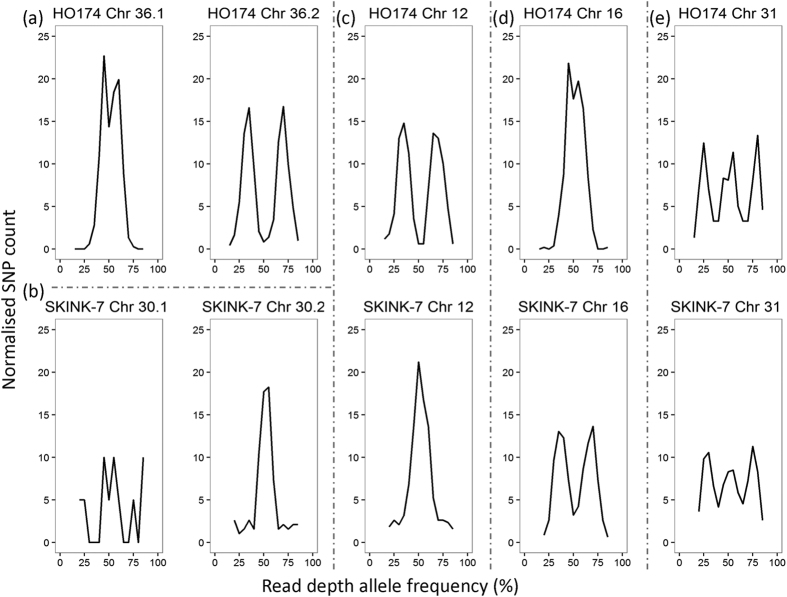
Read depth allele frequency (RDAF) distributions of normalised SNP counts for: (**a**) *L. adleri* HO174 disomic chromosome 36.1 and trisomic chromosome 36.2; (**b**) *L. adleri* SKINK-7 tetrasomic chromosome 30.1 and disomic chromosome 30.2; (**c**) chromosome 12 trisomic in HO174 and disomic in SKINK-7; (**d**) chromosome 16 disomic in HO174 and trisomic in SKINK-7; (**e**) tetrasomic chromosome 31 in HO174 and SKINK-7.

We postulate fission of chromosome 36 for HO174 based on a sharp change in coverage after base 989,698 (chromosome 36.1) with 62-fold median coverage that was 5′ of a gap of unknown length (arbitrarily 100 bp). There was 94-fold median coverage 3′ of this gap from base 989,797 to the chromosome end at 2,589,750 - the remainder of chromosome 36 (36.2) (Fig. 3a). This difference between a disomic chromosome 36.1 and a trisomic chromosome 36.2 was suppported by the median coverage (Fig. 4) and read depth allele frequencies (RDAFs) of heterozygous SNPs (Fig. 5). A large change in coverage was evident when the HO174 reads were mapped to the *L. adleri* (Supplementary Fig. S3) or *L. tarentolae* reference genomes (Supplementary Fig. S4). No changes in coverage were present for SKINK-7 reads mapped the *L. adleri* (Supplementary Fig. S5) or *L. tarentolae* reference genomes (Supplementary Fig. S6), nor for *L. tarentolae* reads mapped to the *L. tarentolae* genome (Supplementary Fig. S7). No HO174 read pairs spanned this location when mapped to the *L. adleri* or the *L. tarentolae* reference genomes (Supplementary Fig. S8), and the same result was found for SKINK-7 (Supplementary Fig. S9). The fission position split two PTUs at a region homologous to LmjF.36.2560-LmjF.36.2570, showed no major change in GC content, and had no excess read coverage symptomatic of a tandem duplication or collapsed repeat (Supplementary Fig. S8, S9). The long length of chromosome 36.2 (1.6 Mb) suggested it was not an amplification such as an episome or a linear mini-chromosome, which would have a higher copy number and would be <740 Kb^53^,^54^. Consequently, this suggests separate chromosomes 36.1 and 36.2 in both *L. adleri* HO174 and SKINK-7.

We propose a second putative fission of chromosome 30 for SKINK-7 based on a marked shift in coverage when the SKINK-7 reads were mapped to *L. adleri* HO174 (Fig. 3b) and *L. tarenolae* (Supplementary Fig. S6). The tetrasomic SKINK-7 chromosome 30.1 spanned *L. adleri* HO174 bases 1–230,911 with 88-fold median coverage, and the disomic chromosome 30.2 at bases 231,011–1,197,246 (the end) had 43-fold median coverage (Supplementary Fig. S5). The change in somy was verified using read coverage (Fig. 4), the RDAFs of heterozygous SNPs (Fig. 5), and was apparent when mapping to the HO174 and *L. tarentolae* reference genomes (Supplementary Fig. S8) because the *L. tarentolae* chromosome also had a gap at the corresponding region (Supplementary Fig. S10). No HO174 or SKINK-7 read pairs spanned the break when mapped to the HO174 reference, and a single SKINK-7 pair crossing the breakpoint when mapped to *L. tarentolae* had a 57 Kb insert size indicating that one read was incorrectly mapped (Supplementary Fig. S9). The chromosome 30 break had no read pile-up and occurred at a contig gap separating PTUs at a region homologous to LmjF.30.0710 (a cell division cycle 16 gene associated with mitosis) and hypothetical gene LmjF.30.0720. These analyses were consistent with a second fission creating chromosomes 30.1 and 30.2 in *L. adleri* SKINK-7 and HO174.

### *L. adleri* is largely disomic but aneuploid

Aneuploidy is an intrinsic feature of *Leishmania* and was measured using read coverage. Disomy was confirmed from a RDAF distribution peak of ~50% using all chromosomal heterozygous SNPs (Supplementary Fig. S11). *L. adleri* HO174 was predominantly disomic (including chromosome 36.1) but ten chromosomes were trisomic (6, 8, 12, 13, 14, 20, 23, 25, 29, 36.2; Fig. 4). Chromosome 2 had a somy of 3.4, symptomatic of a mosaic cell population, and chromosome 31 was tetrasomic, as expected given it was nearly always tetrasomic in sequenced *Leishmania*^34^,^35^,^36^,^37^,^38^,^41^. These estimates were confirmed by the heterozygous SNP RDAF distributions for each chromosome (Supplementary Fig. S12), and were unaffected by GC content bias or local repeats or amplifications (Supplementary Fig. S3).

Repeating this for *L. adleri* SKINK-7 mapped to the HO174 genome suggested less aneuploidy. Only SKINK-7 chromosome 16 was clearly trisomic, chromosome 10 was marginally so, and chromosome 7 was between di- and tri-somy. Chromosomes 30.1 and 31 were tetrasomic (Fig. 4). The heterozygous SNP RDAF distributions for each chromosome confirmed these estimates, except for chromosome 3 whose distribution suggested trisomy, conflicting with the disomy (2.2 copies) indicated by coverage (Supplementary Fig. S13).

Conserved extra chromosomes would have allowed more heterozygous to accrue over time, however there was no difference in the heterozygous SNP rate per 10 Kb segment for SKINK-7 chromosome 30.1 versus 30.2. This was also true for HO174 chromosome 36.1 versus 36.2, suggesting the differences in somy were recent rather than long-term.

### Annotation of the *L. adleri* HO174 reference genome

A total of 7,959 genes were annotated on the *L. adleri* HO174 reference, of which 7,849 were protein-coding (Table 1). 7,570 genes were assigned to chromosomes and 389 to unassigned contigs. 7,845 (98.6%) of the total 7,959 genes were annotated by Companion^55^. Repeating this for the *L. tarentolae* chromosomes and all unassigned contigs (1,351 sequences) produced 7,893 genes, 92.5% of those on *L. tarentolae* TriTryDB v6. A further screen for candidate genes in *L. adleri* HO174 found 117 more, of which 110 had orthologs in *L. major*, one in *L. mexicana*, two in *L. infantum*: four genes without *Leishmania* orthologs encoded hypothetical gene products with homology to other trypanosomatids (Supplementary Table S3).

### Functional and comparative analysis of putative protein-coding orthologous genes

**Figure 6:**
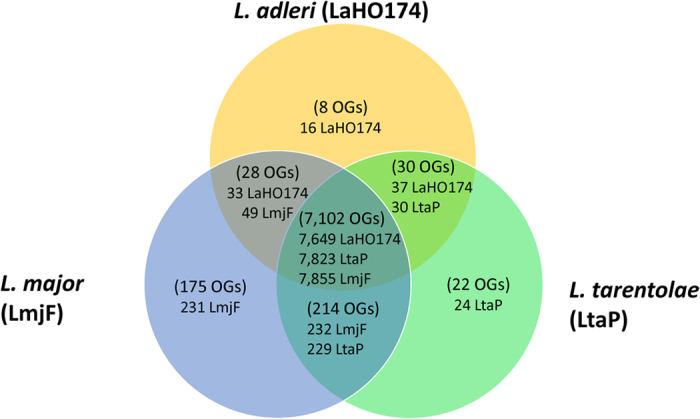
Numbers of genes either unique to or with orthologs in each *L. adleri* HO174, *L. major* Friedlin and *L. tarentaole* Parrot-TarII determined using OrthoMCL v5 orthologous groups (OGs). The OGs are in parentheses. The number of genes in the *L. major* OGs are denoted by LmjF, *L. tarentolae* OGs by LtaP, and *L. adleri* OGs by LaHO174.

The functional differences and composition of protein-coding genes in *L. adleri* were categorised into OGs using OrthoMCL^56^: 7,728 genes (98%) into 7,168 OGs for *L. adleri*; 8,113 (96%) into 7,368 OGs for *L. tarentolae*; and 8,367 into 7,519 OGs for *L. major* (Supplementary Table S4). 98% of *L. adleri* genes had orthologs in *L. major* and *L. tarentolae,* indicating high gene content conservation (Fig. 6). Previously, 250 genes were described as absent in *L. tarentolae* Parrot-TarII but present in *L. major* (Raymond *et al*. 2012). Analysis using OrthoMCLdb v5 and the *L. tarentolae* TriTrypDB v6 proteome found 280 protein-coding genes in 203 OGs absent in *L. tarentolae* Parrot-TarII but present in *L. major* (Supplementary Table S4): 32 had orthologs in *L. adleri* HO174 (Supplementary Table S5).

Of the 16 *L. adleri* genes with no *L. tarentolae* or *L. major* orthologs (Supplementary Table S6), four had orthologs in at least one of *L. infantum, L. mexicana or L. braziliensis*, and three had orthologs in one of the five *Trypanosoma (T. vivax, T. brucei, T. brucei gambiense, T. cruzi* strain CL Brener and *T. congolense*) but not in *L. major, L. infantum, L. mexicana, L. braziliensis* or *L. tarentolae*. Nine had no orthologs in the five *Leishmania* and five *Trypanosoma* listed. Eight of these singletons had domains orthologous to variant-specific surface protein genes in parasites such as *Giardia, Entamoeba* and *Trichomonas vaginalis*, in which their protein products undergo antigenic variation to evade host immune responses and facilitate host adaptation^57^. The closest matches for all eight (35–38% identity) was an unnamed product from *Phytomonas sp.* isolate HART1 - trypanosomatids from this genus can infect plants via an insect vector^58^.

Four of the 32 orthologs in *L. adleri* and *L. major* absent in *L. tarentolae* (Supplementary Table S5) encoded a serine/threonine-protein phosphatase PP1, a folate/biopterin transporter, a protein kinase and DNA polymerase kappa. The nucleoside diphosphate kinase B (LaHO174_323240) gene absent in *L. tarentolae* had five copies in HO174 compared to one in *L. major,* three each in *L. infantum, L. braziliensis* and *L. mexicana*^36^, and two in *L. panamensis* PSC-1^38^. A chromosome 19 gene array encoding autophagy-related protein 8 (ATG8/AUT7/APG8/PAZ2, OG5_137181) involved in endocytic trafficking and recycling^59^ may be absent or partially assembled in *L. tarentolae* because it had two genes, a gap and collapsed repeat (Tables S6 and S8).

### *L. adleri* copy number variation (CNV) and gene arrays

Four of the six large (5.7–19.8 Kb) CNVs in *L. adleri* HO174 were in SKINK-7 (Table 2): one was an amplification of 5.7 Kb including a phosphoglycan beta 1,3 galactosyltransferase 5 gene (SCG5, LaHO174_312750) with five copies in HO174. A 15.9 Kb CNV unique to HO174 on chromosome 27 included three genes with copy numbers of 2.5–3.0: *ABCA8* (LaHO174_271110)*, ABCA9* (LaHO174_271120), and LaHO174_271130 (a cysteine peptidase with a calpain-like domain gene). The sole CNV unique to SKINK-7 was non-coding.

Gene arrays were OGs with haploid copy number of at least two: *L. adleri* had 295 such arrays (Supplementary Table S8), *L. tarentolae* 281 (Supplementary Table S9), *L. major* 289 (Supplementary Table S10), and *L. panamensis* nearly 400, though 285 had just two gene copies (Llanes *et al*. 2015). Collapsed arrays can be detected where the coverage copy number wastwo-fold or more the assembled version by: *L. tarentolae* (119 in Supplementary Table S11) had relatively more than *L. adleri* (62 in Supplementary Table S12). For context, all but twelve arrays in *L. major* were fully resolved (Supplementary Table S13).

## Discussion

A dual strategy combining *de novo* with reference-guided assembly of short DNA sequence reads produced a high-quality draft of the *L. adleri* MARV/ET/1975/HO174 genome isolated originally from a rodent. The HO174 reads were *de novo* assembled into 5,785 scaffolds and contiguated initially into 36 chromosomes using the lizard-infecting *L. tarentolae* Parrot-TarII genome. The final 30.4 Mb *L. adleri* genome has 38 chromosomes with 94.5% of assembled sequence on chromosomes and 69-fold median coverage.

Like all *Leishmania* genomes, it contains tandem arrays of collapsed repeats and genes mapping to multiple chromosomal locations whose true copy number can be inferred using coverage without definite resolution of chromosomal context. Longer reads with greater insert size length variation would map reads more uniquely, enhance contiguation and gene copy number estimates. Despite the inevitable contig gaps, mis-assemblies, and low-quality regions, comparison with other genomes shows that the *L. adleri* genome is largely complete and an asset for understanding the evolutionary basis of host specificity^60^.

The *L. adleri* genome contains 7,959 genes: 7,849 protein-coding ones on 38 chromosomes and 389 on unassigned contigs. Although the vast majority of genes (98.6%) were computationally mapped from reference genomes with perfect matching^55^, visual inspection remains necessary to discover and correct complex gene models. Here, 32 genes were absent in *L. tarentolae* but present in *Leishmania* belonging to other subgenera.

Alignment of seven *L. adleri* HO174 genes with those of 224 other *Leishmania* isolates from infected patients, mammals and insects^49^ showed that HO174 was a *Sauroleishmania* isolate most closely related to *L. adleri* RLAT/KE/1957/SKINK-7 and *L. adleri* RLIZ/KE/1954/1433 compared to *L. tarentolae, L. hoogstraali* and *L. gymnodactyli* (Fig. 1). Consequently, HO174 is the first genome sequenced from the *Sauroleishmania* subgenus isolated from a mammal. Mapping SKINK-7 reads to the HO174 reference also confirmed it as *L. adleri* rather than *L. tarentolae*. Previous MLMT could not classify HO174 clearly^30^ – our work illustrates how genome-wide investigation improves phylogenetic resolution.

The hypothesis of inherent aneuploidy in *Leishmania*^61^ was verified in *Sauroleishmania* HO174, SKINK-7 and Parrot-TarII. The levels of mapped reads and their allelic variants across chromosomes demonstrated that chromosome 36 was split into two portions in *L. adleri* HO174: a disomic chromosome 36.1 (990 Kb) and a trisomic chromosome 36.2 (1,600 Kb, Supplementary Fig. S12). *L. adleri* SKINK-7 had this chromosome 36 fission, but not *L. tarentolae* as indicated previously with long sequence reads^41^. *L. adleri* SKINK-7 chromosome 30 was split into two portions: a disomic chromosome 30.1 (231 Kb) and a trisomic chromosome 30.2 (966 Kb). This was present in HO174 without any somy change.

Most *Leishmania* species have 36 chromosomes, including all members of the *Leishmania* subgenus *–* except for 34 in the *L. mexicana* complex, in which chromosomes 8 and 29 are a fused chromosome 8, and chromosomes 20 and 36 are a fused chromosome 29^36^. In contrast, chromosome 30 is largely conserved as a single unit in trypanosomatids^60^. Chromosomal fission resulting in the truncation of chromosome 4 in *L. tarentolae* LEM115 (365 Kb, similar to Parrot-TarII chromosome 3) isolated from a gecko^2^ was observed during routine subculture in which six out of 20 clones produced a 340 Kb chromosome 4 A^62^. One cloned line had cells with both chromosomes 4 and 4 A in which the chromosome 4 A line outgrew the wild-type after re-cloning, suggesting no fitness loss^62^. Chromosome 4A may have been due to a contraction of the mini-exon gene array, as described for *L. major* chromosome 2^63^. *Trypanosoma cruzi* chromosome 11 had a similar coverage change at a SSR (at 248 Kb) where the segment 5′ of the SSR had a uniformly lower coverage than the 3′ segment. This indicated either loss of the 248 Kb region for one chromosome copy with perhaps partial fixation in the cell population^64^,^65^, or fission into a monosomic chromosome 11.1 and disomic chromosome 11.2.

Chromosomal fission may be more common at SSRs to preserve RNA polymerase II promoters because transcription is initiated at all SSRs independently of PTU context^66^. Elevated acetylation of histone H3 signifies these promoters^67^: *L. major* has high acetylation at positions equivalent to the *L. adleri* chromosome 30 and 36 breaks^66^. Both fission locations coincided with *L. adleri* and *L. major* SSRs: this would yield three SSRs at chromosome 36.1 and two for 36.2 (Fig. 3a). The chromosome 30 break was at a SSR, suggesting 3′ to 5′ transcription of chromosome 30.1 with no SSRs, and two SSRs for 30.2 (Fig. 3b).

Viable new chromosomes must have at least one single origin of DNA replication: and these coincide with SSRs for 30 chromosomes out of 36 in *L. major*^66^. A single bidirectional origin is typically retained after chromosomal fusion in *Leishmania*, such as the single origins on the fused *L. mexicana* chromosomes 8 and 29^66^. *L. major* and *L. mexicana* had chromosome 30 origins at positions equivalent to the *L. adleri* chromosome 30 break, indicating that replication may proceed from this origin 3′−5′ for chromosome 30.1 and 5′−3′ for 30.2. The chromosome 36 origin at bases 1,110,127–1,116,528 in *L. major* was at the 5′ end of the *L. adleri* chromosome 36.2^66^, indicating that a different origin may be used for chromosome 36.1. Consequently, the two fissions may stem from erroneous chromosome replication^68^, and may be functionally neutral, though an additional DNA replication origin on a new chromosome could accelerate cell replication^66^. Alternative to this model, there could be many low-activity origins of replication per chromosome in *Sauroleishmania* as suggested for *Leishmania* promastigotes^69^.

The single early-firing origins on each *L. major* chromosome could represent centromeric regions because they are replicated in early S-phase in other eukaryotes^66^,^70^. The chromosome 30 fission in L. adleri would result in one origin at the 3′ end of chromosome 30.1 and one origin at the 5′ end of chromosome 30.2 based on homology with *L. major*. If a centromere was present at this locus it would be split into two parts, with one functional part on each fission product (centric fission)^71^. The broken chromosome 30 and 36 ends could be protected from degradation through the addition of telomeres, structural rearrangements to protect the chromosomes ends, the development of ring chromosomes through fusion of the broken end with the telomere of the intact end, or translocation of the broken chromosomes onto the end of other intact chromosomes^71^. No evidence of structural rearrangements at the chromosome ends or fission break regions was found here, though telomeric sequences are highly repetitive and were not assembled fully, highlighting a task for future work.

## Conclusions

This study produced the first *Leishmania adleri* high-quality draft genome for the isolate MARV/ET/1975/HO174, which advances the study of the subgenus *Sauroleishmania* of reptile-infecting parasites. We show that short read data can produce comprehensive genome assemblies and allowed for enhanced specimen typing. The discovery of two *L. adleri* chromosome fissions highlights that this feature of genetic diversity may be present in other Trypanosomatid species.

Our results confirm that *L. adleri* HO174 from a well-known mammalian reservoir of *Leishmania* was closely related to other isolates of *L. adleri* originally from lizards. *Sauroleishmania* are not restricted to reptiles, and human-infecting *L. tropica* and *L. donovani* isolates infect lizards, and are likely transferred by *Sergentomyia* from human to lizard^12^. There are abundant zoonotic reservoirs of *Leishmania* including rodents, livestock, mongooses^72^, bats^73^, hyraxes^1^ and dogs. Elimination programmes must treat hosts of any type with high parasitaemia first because they are responsible for most sandfly infections^74^. Given the expansion of *Phlebotomus* and *Sergentomyia* sandfly ranges driven by climate change^75^ and the extensive gene synteny among *Leishmania*, broader testing is required to track isolates from human, livestock and wild hosts in light of the viability of potential interspecies hybrids^76^.

## Methods

### Genomic data sources

MARV/ET/1975/HO174 was isolated from an African grass rat (*Arvicanthis niloticus*) on 24/01/1975. It was received by London School of Hygiene and Tropical Medicine from Liverpool University on 09/09/1980 (Liverpool University cryobank accession LV388). The Wellcome Trust Sanger Institute core sequencing group prepared standard Illumina libraries sequenced by an Illumina HiSeq 2000 v3 platform to generate 18,183,113 paired-end 75 bp reads with a median insert size of 400 (ERX180410).

The *L. tarentolae* RTAR/DZ/1939/Parrot-TarII and *L. major* Friedlin^33^ genomes, protein sequences and annotation (GFF) files were downloaded from TriTrypDB v6. Single-end Illumina shotgun 36 bp reads for Parrot-TarII^41^, 12,680,080 paired-end Illumina Genome Analyzer II 76 bp reads for *L. major* Friedlin (ERX005636)^36^, and 18,322,426 paired-end Illumina HiSeq 2000 100 bp reads for *L. adleri* RLAT/KE/1957/SKINK-7 (aka LRC-L123) (SRX764330)^6^ were analysed.

### Sequence read quality control

The MARV/ET/1975/HO174 library read quality was analysed using FastQC (www.bioinformatics.babraham.ac.uk/projects/fastqc/). PCR primer sequences were removed based on FastQC alignment matches. Potential DNA contaminants were determined where the species of the top hit in alignments with the Non-redundant Nucleotide Database using BLASTn^77^ were not in the *Kinetoplastida* class of the NCBI Taxonomy Database. FastQC was repeated on the decontaminated reads so that reads with a extreme GC content could be removed.

### Genome assembly and improvement

The processed reads were assembled *de novo* using Velvet v1.2.09^43^ with kmer of 53, expected kmer coverage of 16 and coverage threshold of eight. A kmer of 53 maximised the N50 for contigs >100 bp. These contigs were scaffolded with SSPACE v2.0^44^ because it has previously produced output with fewer scaffolds and longer N50s than Abyss or SOAP^44^. Gaps were closed using Gapfiller for ten iterations^45^. Erroneous bases were corrected by re-mapping reads to the scaffolds using iCORN for ten iterations^46^.

Putative mis-assemblies were screened by splitting scaffolds at potential errors with REAPR^47^, which evaluated the N50, corrected N50 and the percentage of EFBs after mapping the initial reads to the initial assembly (Supplementary Table S1). EFBs had five or more correctly-oriented read pairs, matched the expected insert size, had no mismatches and a small fragment coverage distribution error^47^. Scaffold structure and read pair mapping were visually examined using IGV v2.3^78^.

The scaffolds were contiguated into 36 chromosomes using the *L. tarentolae* genome as a reference with ABACAS^48^. Gaps >100 bp were shortened to 100 bp and unplaced contigs <1 Kb were removed. Unplaced bin contigs were aligned using MegaBLAST against a database of 753 minicircle and 152 maxicircle kDNA sequences obtained from Genbank^76^. Contigs with E value <0.01, bitscore >100 and identity >40% were annotated as minicircle or maxicircle kDNA. The final 38 HO174 chromosomes were verified visually by alignment with those for *L. tarentolae* using the Artemis Comparison Tool (ACT)^79^.

### Phylogenomic characterisation

The genus and species of the HO174 genome was assessed using seven genes from 222 published isolates^49^. Reads for SKINK-7 were assembled into contigs using Velvet^43^ with kmer of 53 yielding an assembly with 15,507 contigs and N50 of 4.88 Kb. Orthologous genes were extracted from the HO174 genome, *L. tarentolae* genome^41^ and SKINK-7 assembly using BLASTn and aligned with the 222 using Clustal Omega v1.1^50^. A neighbour-net network of uncorrected p-distances was constructed using SplitsTree v4.13.1^51^. To pinpoint the phylogenetic position of HO174 within the *L. tarentolae* complex, the two genes of five *Sauroleishmania* species^7^ were aligned with the HO174 orthologs as above - the phylogenetic structure of each gene was similar (Supplementary Fig. S14).

### Gene annotation

The HO174 chromosomes and contigs were annotated by transferring gene models from the *L. major* genome to HO174 using species-level transfer with RATT^80^ through Companion^55^. This system used *ab initio* gene-finding by Augustus trained on *L. major*, and predicted tRNA, rRNA and ncRNA genes using Infernal^81^ and Aragorn^82^. Open reading frames >450 bp identified by Artemis^83^ were screened for genes missed by Companion. Putative open reading frames with potential start and stop codons were aligned with the NCBI protein database using BLASTp, where those with an E-value <0.1 and identity >30% were considered as valid coding sequences for manual examination using Artemis and ACT^78^. This manual correction was used where a gene extended over a gap of unknown length to trim it to the edge of the first gap, and similarly genes with multiple stop codons were adjusted to the first stop codon.

### Estimating chromosome copy number

To calculate the chromosome copy number based on coverage, reads were mapped using SMALT v5.7 (www.sanger.ac.uk/resources/software/smalt/) with parameters set to exhaustive mapping and a maximum insert size of 1000. The reference genomes against which reads were mapped were indexed with a kmer of 13 and step of two. Duplicate reads were removed using Samtools rmdup^84^ and coverage at each base was retrieved using Bedtools ‘genomecov’ v2.17.0^85^. For each chromosome, we calculated the median read coverage. Assuming that most chromosomes were disomic, the median of these chromosomal values produces a reliable estimate of the coverage of disomic chromosomal coverage, and so dividing it by two gives the haploid value. Thus, the copy number of each chromosome was estimated as the chromosome’s median coverage divided by this haploid value. Chromosome copy numbers were visually confirmed using the RDAF distribution of heterozygous SNPs generated with R packages ggplot2 and gridExtra. RDAFs obtained from Samtools pileup v0.1.11 were binned with a step of 0.05 from 0.15 to 0.85: values outside this range are uninformative in the context of distinguishing somy of up to five, and likely represented sequencing artefacts.

### Detection of CNVs

CNVs were examined at non-masked regions using the same coverage-based approach used for chromosome copy numbers. The median haploid copy numbers of non-overlapping 10 Kb blocks were estimated for uniquely mapped reads with mapping quality >30 using Samtools view^84^ and Bedtools ‘makewindows’ v2.17.0^85^. CNVs were denoted as regions with a two-fold or greater change over the chromosomal median coverage, and were verified visually with ggplot2 and IGV^78^. The copy number of each *L. major, L. tarentolae* and *L. adleri* gene was estimated without removing non-uniquely mapped reads, which could bias estimates due to lower coverage at genes with multiple homologs. HO174 reads were mapped to HO174 with chromosome 36 as a single unit and then again with it split into chromosomes 36.1 and 36.2 at the breakpoint to resolve gene copy number more accurately. Similarly, SKINK-7 reads were mapped to *L. adleri* HO174 with chromosome 30 as a single block and split into chromosomes 30.1 and 30.2.

### Single-nucleotide variant discovery

Repetitive sequences, low-quality regions, homopolymers and small tandem repeats discovered using Tantan v0.13^86^, segments within 300 bases of contig edges, and regions within 100 bases of gaps were masked to exclude false SNPs. Candidate SNPs were screened with Vcftools v0.1.12b^87^, Samtools mpileup v0.1.18, Bcftools v0.1.17-dev, and the Samtools 0.1.18 samtools.pl varFilter^84^ where they had: base quality >25; mapping quality >30; coverage >5 and <100; SNP quality >30; a non-reference RDAF >0.1; a forward-reverse read coverage ratio >0.1 and <0.9; 2+ forward reads, and 2+ reverse reads. SNPs were considered heterozygous if the RDAF >0.1, and homozygous if the RDAF >0.85.

### Identification of orthologous groups (OGs) and gene arrays

*L. adleri* and *L. tarentolae* proteins were assigned to OrthoMCL OGs using the OrthoMCLdb v5 webserver. This excluded 44 *L. adleri* genes classified as pseudogenes by Companion: subsequent manual correction indicated these were valid protein coding genes. The results were compared with 11,825 OGs retrieved from OrthoMCLdb release 5^56^ for those in at least one of: *L. major* strain Friedlin, *L. infantum, L. braziliensis, L. mexicana, T. vivax, T. brucei, T. brucei gambiense, T. cruzi* strain CL Brener and *T. congolense*. 7,654 of these OGs were present in at least one of: *L. braziliensis, L. infantum, L. major* or *L. mexicana*. The copy number of each OG in *L. tarentolae, L. major* and *L. adleri* was estimated from the haploid read coverage of each gene in the OG and summing across all the genes in the OG. Gene arrays were defined as segments containing 2+ haploid gene copies with the same OrthoMCL identifier, and so could be located in cis or trans. Large arrays (10+ gene copies) in *L. major, L. adleri* HO174 and *L. tarentolae* were examined and those with unassembled copies were identified by finding arrays with a haploid copy number more than twice the assembled gene number.

### Availability of materials and data

The data sets for *Leishmania adleri* MARV/ET/1975/HO174 are:

[1]DNA read data available with accession number ERX180410 at the NCBI Sequence Read Archive http://www.ncbi.nlm.nih.gov/sra/ERX180410 and European Nucleotide Archive http://www.ebi.ac.uk/ena/data/view/ERX180410.

[2]The BioProject PRJEB17628 consensus genome sequence FASTA file at https://figshare.com/articles/L_alderi_HO174_genome_FASTA_file/4645450 and annotation EMBL file at https://figshare.com/articles/L_adleri_HO174_genome_annotation_EMBL_file/4645477.



## Additional Information

**How to cite this article**: Coughlan, S. *et al*. The genome of *Leishmania adleri* from a mammalian host highlights chromosome fission in *Sauroleishmania. Sci. Rep.* **7**, 43747; doi: 10.1038/srep43747 (2017).

**Publisher's note:** Springer Nature remains neutral with regard to jurisdictional claims in published maps and institutional affiliations.

## Supplementary Material

Supplementary Information

Supplementary Tables

## Figures and Tables

**Table 1:** Summary statistics for the *L. adleri* and *L. tarentolae* genomes, including unassigned (bin) contigs for both.

Genome statistics	*L. adleri* HO174	*L. tarentolae* ParrotII
Number of chromosomes	38	36
All genes	7,959	8,530
Protein coding genes	7,849	8,454
Genes on chromosomes	7,570	8,282
Genes on bin contigs	389	248
Number of gaps	4,350	4,568
N content (%)	0.64	3.77
Chromosomes total length (bp)	28,686,960	31,056,039
Bin sequence total length (bp)	1,664,372	578,687
Genome length (bp)	30,351,332	31,634,726
GC content (%)	56.76	56.66
Median Coverage	69	30

*L. adleri* has two additional chromosomes due to the fission of chromosome 30 in to 30.1 and 30.2 and chromosome 36 into chromosome 36.1 and chromosome 36.2.

**Table 2:** Amplified genes in *L. adleri* HO174 (top) and *L. adleri* SKINK-7 (bottom).

*L. adleri* HO174							
Copy number variant information						Gene information	
Chr	Copy number	Start	End	Length (bp)	Gene number	Gene ID	Gene product
10	2	490,001	500,889	10,888	3	LaHO174_101360	Phosphate-repressible phosphate permease-like protein
						LaHO174_101370	Pteridine transporter (folate/biopterin transporter)
						LaHO174_101380	Delta-12 fatty acid desaturase
17	5.5	22,673	42,500	19,827	4	LaHO174_170090	Elongation factor 1 -alpha
						LaHO174_170100	Receptor-type adenylate cyclase a
						LaHO174_170110	Receptor-type adenylate cyclase b
						LaHO174_170120	Receptor-type adenylate cyclase
26	3.5	931,670	941,785	10,115	3	LaHO174_262430	Protein kinase
						LaHO174_262440	Conserved hypothetical protein
						LaHO174_262450	Paraquat-inducible protein-A (PqiA)
27*	2.4	437,425	453,344	15,919	3	LaHO174_271110	ATP-binding cassette subfamily A, member 8 (ABCA8)
						LaHO174_271120	ATP-binding cassette subfamily A, member 9 (ABCA9)
						LaHO174_271130	Cysteine peptidase, Clan CA, family C2
31	3.9	1,181,426	1,187,096	5,670	2	LaHO174_312740	Conserved hypothetical protein
						LaHO174_312750	Phosphoglycan beta 1,3 galactosyltransferase 5
33*	2.2	1,034,500	1,040,973	6,473	None		
***L. adleri*****SKINK-7**							
10	2.5	490,001	500,889	10,888	2	LaHO174_101360	Phosphate-repressible phosphate permease-like protein
						LaHO174_101370	Pteridine transporter (folate/biopterin transporter)
						LaHO174_101380	Delta-12 fatty acid desaturase
17	3.6	22,673	42,500	19,827	2	LaHO174_170090	Elongation factor 1 -alpha
						LaHO174_170100	Receptor-type adenylate cyclase a
						LaHO174_170110	Receptor-type adenylate cyclase b
						LaHO174_170120	Receptor-type adenylate cyclase
17*	2	518,715	521,099	2,384	None		
26	2.9	931,670	941,785	10,115	3	LaHO174_262430	Protein kinase
						LaHO174_262440	Conserved hypothetical protein
						LaHO174_262450	Paraquat-inducible protein-A (*PqiA*)
31	3.34	1,181,426	1,187,096	5,670	4	LaHO174_312740	Conserved hypothetical protein
						LaHO174_312750	Phosphoglycan beta 1,3 galactosyltransferase 5

*CNVs unique to each strain.
