# Supplementary material for: The genome of Leishmania adleri from a mammalian host highlights chromosome fission in Sauroleishmania
Source: Sci Rep. 2017 Mar 3;7:43747. doi: 10.1038/srep43747 (PMC5335649; doi:10.1038/srep43747)
Supplement: Supplementary Information [file srep43747-s1.doc]

**Title:** The genome of *Leishmania adleri* from a mammalian host highlights chromosome fission in *Sauroleishmania*

Simone Coughlan (1), Peter Mulhair (2), Mandy Sanders (3), Gabriele Schonian (4), James A. Cotton (3), Tim Downing (1,2)*.

(1) School of Mathematics, Applied Mathematics and Statistics, National University of Ireland, Galway, Ireland.

(2) School of Biotechnology, Dublin City University, Dublin, Ireland.

(3) Wellcome Trust Sanger Institute, Hinxton, UK.

(4) Charite University Medicine, Berlin, Germany.

**Supplementary Results**

**Annotation**

82 of the RNA genes were tRNAs: only one fewer than *L. major* (Ivens et al. 2005). 14 of these 82 were rRNAs, five were small nucleolar RNA (snoRNA) genes and two were small nuclear RNA (snRNA) genes. Seven pseudogenes were annotated. The number of snoRNA genes predicted was much lower than that predicted for other *Leishmania* such as *L. major* Friedlin, *L. infantum* JPCM5*, L. panamensis* PSC-1 and *L.* *braziliensis* M2904 which have 741, 50, 54 and 30, respectively (Ivens et al. 2005; Peacock et al. 2007; Llanes et al. 2015; Rogers et al. 2011). SnoRNA genes were not annotated in *L. tarentolae* but Companion only predicted two. A test run using the Companion webserver with the published *L. braziliensis* M2904 genome with itself as a reference only finds ten snoRNAs (rather than 53): repeating this for *L. panamensis* PSC-1 using *L. braziliensis* M2904 as a reference finds just three snoRNA genes (rather than 30).

Most of the 117 manually discovered genes encoded hypothetical gene products, but two were ATP-binding cassette (ABC) gene family members: a duplicate *ABCA9* homolog (LaHO174_270850) and *ABCC2* (LaHO174_230250).

Over-represented gene ontology (GO) terms among *L. adleri* HO174 protein-coding genes were screened using GOseq v1.18 (Young et al. 2010) such that terms with Benjamini-Hochberg-corrected P<0.1 were considered over-represented. No over-represented GO terms were identified among chromosome 30.1, 30.2, 36.1 or 36.2 protein-coding genes.

Unassigned contig 01 contains *ABCB4* with orthologs on chr34 in other *Leishmania*. This gene was homologous to the multidrug resistance 1 gene (MDR-1), which has been associated with resistance to 5-fluorouracil in *L. amazonensis* promastigotes (Katakura et al. 2004), to topoisomerase-binding daunorubicin, and to tubulin-binding vinblastine (Ashutosh, Sundar, and Goyal 2007).

**Reference masking and SNP calling**

For the *L. adleri* HO174, a total of 2,908,704sites were masked out of results (1,865,904 repetitive bases called by tantan, 172,800 potential low quality bases around gaps and 870,000 potential low quality bases at sequence edges). For the *L. tarentolae* genome a total of 3,694,196 sites were masked out (1,975,331 from tantan, 810,600 sites near sequence edges and 908,265 sites at gap edges). Only five of 10,663 SNPs detected using self mapped *L. adleri* HO174 reads were homozygous (RDAF >=0.85) and 10,658 SNPs were heterozygous indicating that most bases in the assembly are correct. SNPs were not examined for *L. tarentolae* because the read quality scores were not available.

**Phylogeny construction**

The seven genes were (El Baidouri et al. 2013): elongation initiation factor 2 alpha subunit (LaHO174_030250), spermidine synthase 1 (LaHO174_040600), zinc binding dehydrogenase-like protein (LaHO174_100590), translation initiation factor alpha subunit (LaHO174_120010), nucleoside hydrolase-like protein (LaHO174_140120), RNA polymerase II (LaHO174_312240) and a hypothetical gene (LaHO174_310180).

**Genes in *L. tarentolae* and *L. adleri* but absent *in L. major***

Of the 30 *L. adleri* genes with orthologs in *L. tarentolae* but not in *L.major*, 18 had orthologs in another *Leishmania* (Supplementary Table S14). 12 *L. adleri* genes had orthlogs in *L. tarentolae* that were absent from *L. major*, *braziliensis, mexicana* and *infantum*, but were present in at least one of six *Trypanosoma* genomes. Among these 12, one encodes an anaphase promoting complex or cyclosome (APC/C) subunit 11 (LaHO174_160590) in the APC/C, a multi-subunit E3 ubiquitin ligase that marks cell cycle regulator proteins and mitotic cyclins for degradation by the proteasome and helps initiate the metaphase-anaphase transition to exit mitosis (Eme *et al.* 2011). APC acivity is regulated by phosphorylation and regulatory proteins CDC20 and CDH1. *Cdc20* homologs are present in *L. donovani*, *L. infantum* and *L. major* (Listovsk*y et a*l. 2011) and several APC subunit homologs have previously been identified in *T. brucei* including *Apc11* (Bessat et al. 2013). A cell division cycle protein 20 (*Cdc20*) gene is also at chr24 in both *L. adleri and L. tarentolae* (LaHO174_241770 and LtaP24.1870) indicating that ubiquitin-dependent proteasomal degradation may be involved in their cell cycles.

**Genes in both *L. adleri* and *L. major* with no orthologs in *L. tarentolae***

No *Ndk* gene was present in *L. tarentolae*, but it had five copies in *L. adleri* HO174 (*Ndk B,* LaHO174_323240). Its protein product catalyses phosphate transfer during NTP homeostasis (Kolli et al. 2008) and participates in purine salvage pathways in trypanosomatids (Landfea*r et a*l. 2004). It assists macrophage infection by *L. amazonensis* LV78 though preventing ATP-mediated lysis of host macrophages, preserving the cells for use by *Leishmania* (Kolli et al. 2008). Promastigotes progressively release NDK leading to its accumulation in stationary phase, when they become metacyclic. Concordant with this, *ndk* expression was up-regulated in *L. major* metacyclic promastigote and amastigote stages (Almeida et al. 2004) and *L. mexicana* promastigotes (Holzer, McMaster, and Forney 2006), though it was down-regulated in antimony-resistant *L. infantum* Sb2000.1 promastigotes (Leprohon et al. 2009).

The calrectulin gene (LaHO174_312230) encodes a chaperone involved the retention of misfolded proteins targeted for proteasome degradation: this was absent in *L. tarentolae* but present as a single copy in *L. adleri* and other *Leishmania* genomes. Overexpression of a *L. donovani* truncated calreticulin ortholog resulted in decreased secretion of acid phosphatase glycoproteins and a reduction in parasite survival inside macrophages, suggesting with a role in virulence-associated protein transfer in the secretory pathway (Debrabant et al. 2002) as an amastigote (McNicoll et al. 2006).

**Genes exclusive to *L. tarentolae***

A total of 24 assembled genes were absent in *L. adleri* and *L. major* but present in *L. tarentolae* (Supplementary Table S15)*.* Ten were present in at least one of *L. infantum***,** *L. braziliensis* or *L. mexicana*. An amastin-like surface protein with one assembled copy had four copies in *L. tarentolae.* 14 *L. tarentolae* genes were exclusive to *L. tarentolae*, ten of these have orthologs in *Trypanosoma* and include two GP63 pseudogenes (LtaP10.0550 and LtaP10.0570), an expression site-associated gene (LtaP24.1490), a gene for a zinc-finger protein (LtaP26.0070) and a gene encoding a high cysteine membrane protein group 2 with orthologs in *Giardia* (LtaP11.1290). Four other genes encoding surface antigen-like protein (LtaP11.1290), malate dehydrogenase (LtaP34.0490) and Ser/Thr protein phosphatase family proteins (LtaP34.0940 and LtaP34.0890) had no *Leishmania* or *Trypanosoma* orthologs. One gene, LtaP27.2450 which also had eight orthologs in *T. congolense* (OG5_158273) had 30 haploid copies in *L. tarentolae* (Supplementary Table S15) but no domains were discovered.

**Gene arrays in *L. tarentolae***

The higher number of OGs previously reported for *L. tarentolae* at 7,449 (Raymond et al. 2012) was likely due to the amalgamation of previously separate OGs in OrthoMCL v2 compared with v5. There were 30 predicted copies of genes (13 assembled copies) coding for hypothetical proteins in one array in *L. adleri* (OG5_139233) and a staggering 156 copies of *L. tarentolae* genes predicted in the same array (51 assembled copies) with only two haploid copies predicted for *L. major*. The proteins in this array typically contain leucine-rich repeat (LRR) domains (Interpro ID: IPR032675), and some have growth factor receptor cysteine-rich domains (Interpro ID: IPR009030). The latter are found in a range of eukaryotic proteins involved in signal transduction by receptor tyrosine kinase. LRR domains are generally 2-29 amino acids long, contain 2-45 motifs, and provide a structural framework for protein-protein interactions (Kobe and Kajava 2001). LRRs are the largest repeat class found in *Leishmania* (Peacock et al. 2007) and are involved in interactions between macrophage complement receptors and the parasite surface (Kedzierski et al. 2004).

***L. adleri* copy number variation (CNV)**

Alpha- and beta-tubulin genes had much higher copy numbers in *L. adleri* (18 alpha- and 17 beta-tubulin copies), *L. major* (10 and 18), *L. mexicana* (6 and 7, 36), *L. panamensis* PSC-1 (21 and 32) compared with *L. tarentolae* Parrot-TarII (0 and 2). There was a gap at the Parrot-TarII alpha-tubulin gene locus but these genes were present in other *L. tarentolae*, suggesting these regions may not be completely assembled in Parrot-TarII. The structural subunit of microtubules is the α/β heterodimer formed from these tubulins. Microtubules dictate cell shape, flagellar motility, intracellular transport, are drug targets, and interact with ATG8 to help deliver autophagosomes to the vacuole/endosomal-lysosomal compartment (Williams et al. 2006). This assists with nutrient recycling through autophagy (Williams et al. 2009; Williams et al. 2012), which is crucial for life cycle stage differentiation and surviving nutrient-limiting conditions (Williams et al. 2009). Here, an *ATG8* gene array was absent in *L. tarentolae* but present in *L. major* and *L. adleri*, so these tubulin and *ATG8* gene family changes may interact to promote stress tolerance.

A 10.9 Kb-long chr10 CNV with 2-3 copies in HO174 and SKINK-7 spanned three genes encoding a phosphate-repressible phosphate permease-like protein, a pteridine transporter and a delta-12 fatty acid desaturase (Table 2). *Leishmania* parasites are pteridine auxotrophs so multiple transporters help transfer it from the insect or host (Ouellette et al. 2002; Vickers and Beverley 2011). The antifolate drug methotrexate, used in *leishmania* treatment, targets that folate biosynthesis pathway to inhibit cellular growth (Ouellette et al. 2002). These include transmembrane pteridine transporters, and associated proteins like pteridine reductase are potential drug targets (Kaur et al. 2011). The pteridine transporter protein amplified here has a BT1 domain and both the BT1 locus and pteridine reductase 1 (PTR1) can undergo amplification either spontaneously or after selection for anti-folate resistance, as either extra-chromosomal linear or circular elements. However, the BT-1 gene itself (LaHO174_354810) is not amplified here. Amplifications and mutations of other folate transporter genes may be driven by drug pressure or pteridine limitation (Vickers and Beverley 2011).

The gene with the highest copy number in *L. adleri* was elongation factor 1-alpha (*EF-1α*, LaHO174_170090), which had 53 copies compared to 38 in *L. tarentolae* and 22 in *L. major* (21 were reported in Rogers et al. (2011)). Only a single *EF-1α* reference copy was in the *L. adleri* and *L. tarentolae* genomes, relative to seven in *L. major. EF-1α* genes undergo extensive changes in copy number in *Leishmania*, including *L. infantum* JPCM5 (7 to 12 copies), *L. braziliensis* M2904 (1 to 10) (Rogers et al. 2011), and *L. panamensis* PSC-1 (1 to 15) (Llanes et al. 2015). Here, it was amplified in both HO174 (5-6 copies) and SKINK-7 (four copies). *EF-1α* encodes a highly conserved GTP-binding protein involved in protein translation, and is a candidate virulence factor in *L. donovani* because it deactivates infected macrophage cells by binding and stimulating the host Src homology 2 domain containing tyrosine phosphatase-1 (SHP-1) (Nandan et al. 2002). This activation of host SHP-1 is associated with leishmaniasis disease pathogenesis, and SHP-1 is inhibited by sodium stibogluconate (Leifso et al. 2007). *EF-1α* is up-regulated in antimony-resistant *L. infantum* promastigotes compared with wild type (Brotherto*n et a*l. 2013). A *L. donovani* EF-1α homolog (LdBPK_354220) had a reduced copy number in a sample with resistance to antimonials during miltefosine-resistance induction (Shaw et al. 2016).

*EF-1α* was within an amplified 19.8 Kb region with three other genes (Table 2), all receptor-type adenylate cyclases (A, B and a putative one). Receptor type adenylate cyclases were down-regulated during the metacyclic promastigote-to-amastigote transition in *L. major* (Dillon et al. 2015). *L. adleri* HO174 had 13 copies of the OG containing the receptor type adenylate cyclase genes compared with eight for *L. tarentolae*, six for *L. major,* five for *L. infantum*, two for *L. mexicana*, and seven each for *L. panamensis* (Llanes et al. 2015) and *L. braziliensis* M2904 (Rogers et al. 2011).

*L. tarentolae* read coverage levels indicated 84 leishmanolysin (aka GP63) genes, whereas *L. adleri* had 37, more similar to the 31 in *L. braziliensis,* 15 in *L. infantum* and 13 in *L. mexicana,* six in *L. major* (five in and 28 in *L. panamensis* (Llanes et al. 2015). 49 GP63 genes were reported in *L. tarentolae* (Raymond et al. 2012), possibly due to differences in OrthoMCL OG assignment or the addition of 253 protein-coding genes in the TriTryDB v6 genome. Leishmanolysin is a cell surface zinc-dependent metalloprotease and virulence factor involved in cleaving the VAMP8 membrane fusion regulator to evade phagocytosis by host macrophages (Casgrain et al. 2016). Leishmanolysin is up-regulated during the promastigote stage (Yao, Donelson, and Wilson 2003) and metacyclic promastigote-to-amastigote transition (Dillon et al. 2015).

***L. adleri* ABC gene family evolution**

The ABC protein superfamily has eight families (A-H). The chr27 CNV unique to HO174 contained an amplification of *ABCA8* and *ABCA9* orthologs. ABCA8 assists with lipid translocation and is implicated in infection (Araujo-Santos et al. 2005). Previously, other ABCA subfamily members like ABCA3 were overexpressed in antimony-resistant *Leishmania* (Leprohon et al. 2006). The other gene within this CNV encodes a cysteine peptidase with a calpain-like domain. Calpains assist cytoskeletal remodelling and signal transduction during differentiation (Mottram, Coombs, and Alexander 2004) and inhibiting them is a drug target (d'Avila-Levy et al. 2006). Orthologs of this gene were up-regulated in log-phase *L. major* and *L. infantum* promastigotes *in vitro* (Rochette et al. 2008) as well as in in antimony-resistant *L. infantum* promastigotes (Brotherton et al. 2013). Cysteine proteases are essential for pathogenicity in mammalian hosts in *L. tropica* (Mahmoudzadeh-Niknam and McKerrow 2004) and suppress host protective immune responses during *L. mexicana* infection (Buxbaum et al. 2003). They also regulate leishmanolysin expression through endocytic SNARE proteins VAMP3 and VAMP8, and thus affect parasitophorous vacuole formation (Matte et al. 2016).

In *L. adleri* HO174, a region homologous to the *ABCC9* gene may have lost function because it contains two genes: one had high homology to a RNA polymerase Rpc34 subunit gene (LmjF.29.1320), and the other has to a serine-threonine protein kinase gene (LmjF.29.1320). *L. donovani* amastigotes with a *ABCC9* (LdBPK.24.1510) deletion had faster growth and increased virulence in mice (Zhang and Matlashewski 2012). An *ABCC9* ortholog is present in *L. mexicana* as well, but is absent in *L. major* and *Viannia* (Zhang and Matlashewski 2012), and although the C-terminus has homology to a *L. tarentolae* gene (LtaP.24.1590), the N-terminus is pseudogenised, suggesting a truncated product.

A novel ORF for an *ABCC2* (chr23:69,197-71,908) copy had high homology to a P-glycoprotein gene (LmjF.23.0220): ABCC2 decreases the accumulation of flavonoid baicalein in *L. donovani* as LdMRP2 (Chowdhury et al. 2014) and the P-glycoprotein has a well-established role in drug resistance. In addition, ABCC3 (P-glycoprotein A) is associated with antimony-resistance in *L. tarentolae* (Haimeur et al. 2000), as has ABCC4 (P-glycoprotein E) with methotrexate-resistance (Gamarro et al. 1994), ABCC7 (pentamidine resistance protein 1) with pentamidine resistance (Coelho *et al.* 2003), and ABCC3/4/5/7 with antimonials (Leprohon, Legare, and Ouellette 2009).

**Somy in *L. tarentolae***

Chromosomes 18, 22, 29, 31 and 32 are predicted to be trisomic and chromosomes 3, 5, 6, 13 and 16 exhibit intermediate depths (ploidy estimates of 2.5, 2.6, 2.7, 2.7 and 2.6 each). Interestingly, chromosome 31 was predicted to be trisomic and not tetrasomic in *L. tarentolae*. Trisomy of chromosome 31 has only previously been reported in the predominantly diploid *L. donovani* LV9 (MHOM/ET/1967/HU3) strain (Rogers et al. 2011) and *L. peruviana* LEM-1537 (Valdivia et al. 2015). Mosaic aneuploidy was evident in each sample, consistent with the presence of genetic heterogeneity in the cell population.

***L. adleri* heat-shock protein (*hsp*) gene evolution**

Variation at *hsp70* has been associated with the resistance to antimony in *L. tarentolae* (Brochu, Haimeur, and Ouellette 2004) and can be used for species identification (Van der Auwera et al. 2013). *Hsp70* orthologs LtaP29.1400 and LaHO174_291400 were homologous to a highly conserved hypothetical gene (LmjF.29.1240) at 490,853-3,174 with *hsp70* activity based on its N-terminus nucleotide-binding domain. LaHO174_291400 initially mapped to 474,995-7,175, however it has an N-terminal extension of 171 bases corresponding to 57 amino acids starting at 474,824, producing a putative polypeptide of 726 amino acids. LtaP29.1400 has no start codon for this N-terminal extension. Like LmjF.29.1240 and LtaP29.1400, LaHO174_291400 also contains three inferred structural tetratricopeptide repeat encompassing a protein-binding domain at its C-terminus.

**Changes in *Sauroleishmania* gene dosage may affect phosphoglycan expression**

The cell surface lipid composition of *L. adleri* is similar to that of other pathogenic members of the *Leishmania* and *Viannia* subgenera for glycoinositolphospholipids but not lipophosphoglycans (LPGs). LPGs affect promastigote adhesion to lectins in the sandfly midgut epithelium, and detachment of metacyclic promastigotes from the midgut. They moderate host specificity by stopping phagolysosome biogenesis, changing antioxidant resistance (Lodge and Descoteaux 2008), and the host innate immune response (Aebischer et al. 2005; Becker et al. 2003). Here, HO174 had 5.4 copies of a gene encoding SCG5 (LaHO174_312750) compared to just 1.2 copies in *L. tarentolae* and 1.0 in *L. major*: its product helps modify phosphoglycan repeats for cell surface LPGs.

**Supplementary Figures**

**
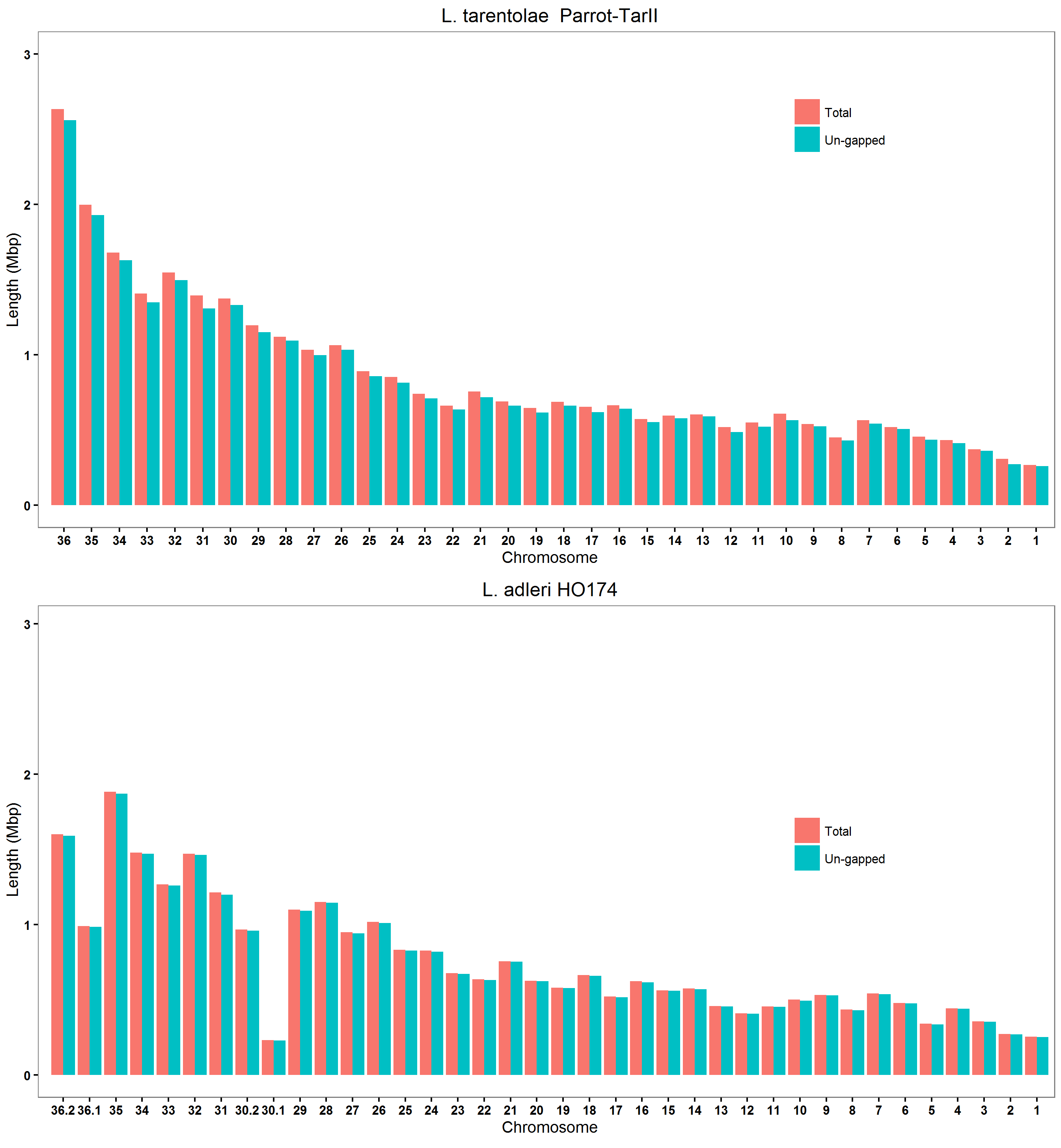
**

Fig. S1: Chromosome lengths of *L. tarentolae* and *L. adleri* HO174 showing lengths with gaps included (red) and lengths with gaps excluded (green).


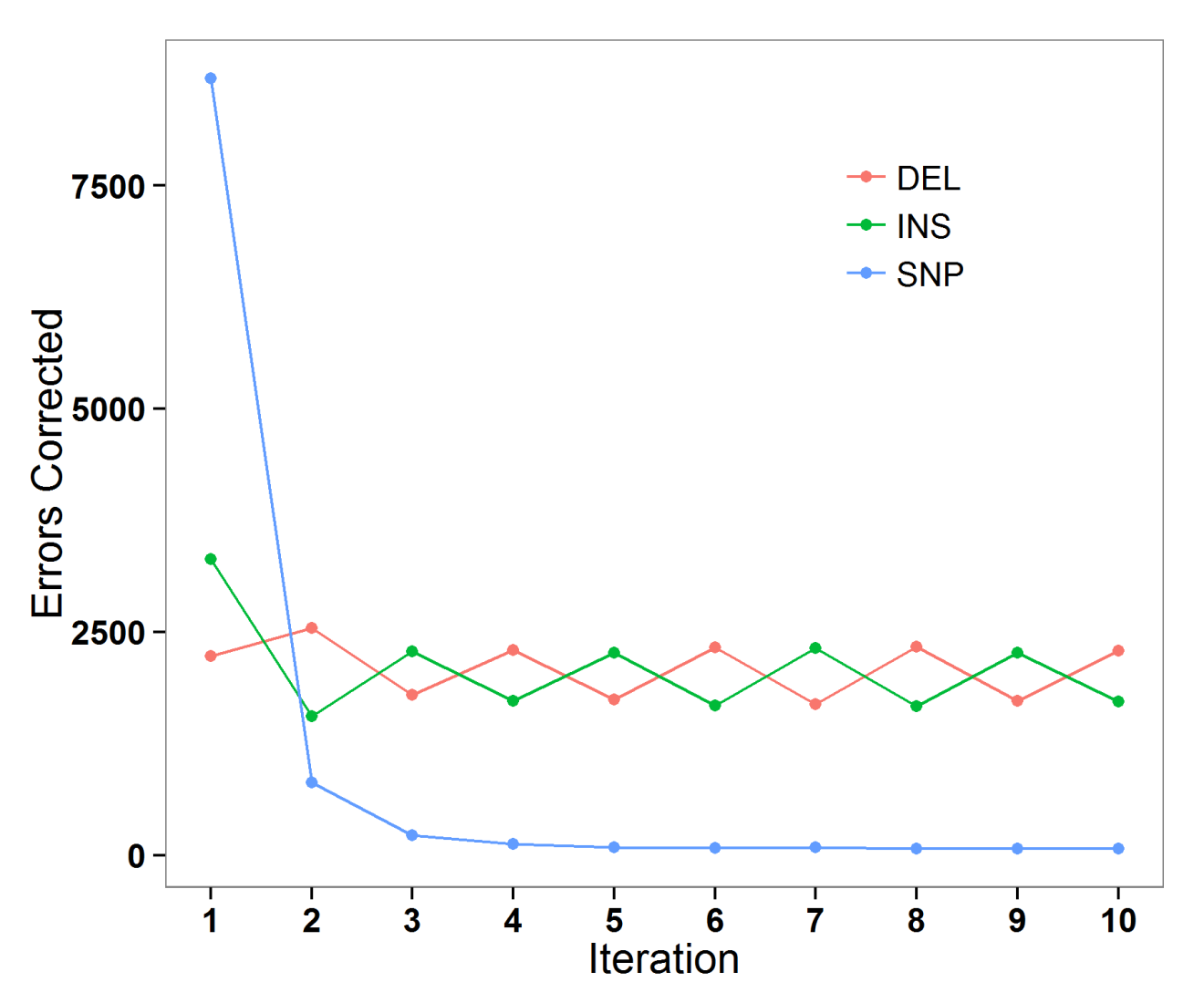


Fig. S2: Number of iCORN corrections over 10 iterations.


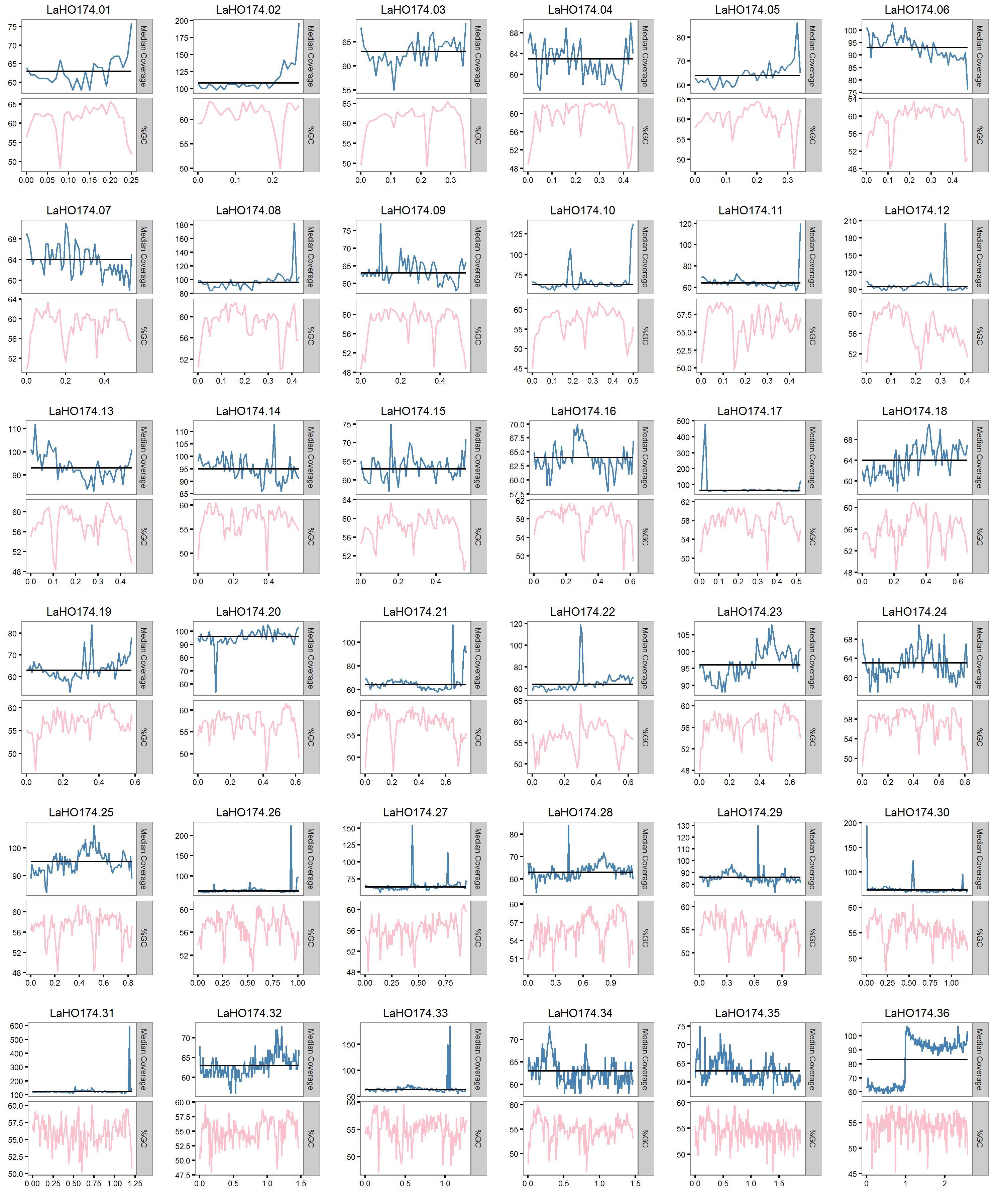


Fig. S3: Median coverage of *L. adleri* HO174 mapped to itself with unbroken chromosomes 30 and 36 measured in 10 Kb intervals (blue lines) for each chromosome. Black lines indicate median chromosomal coverage for that chromosome and pink lines show GC content in 10 Kb intervals.


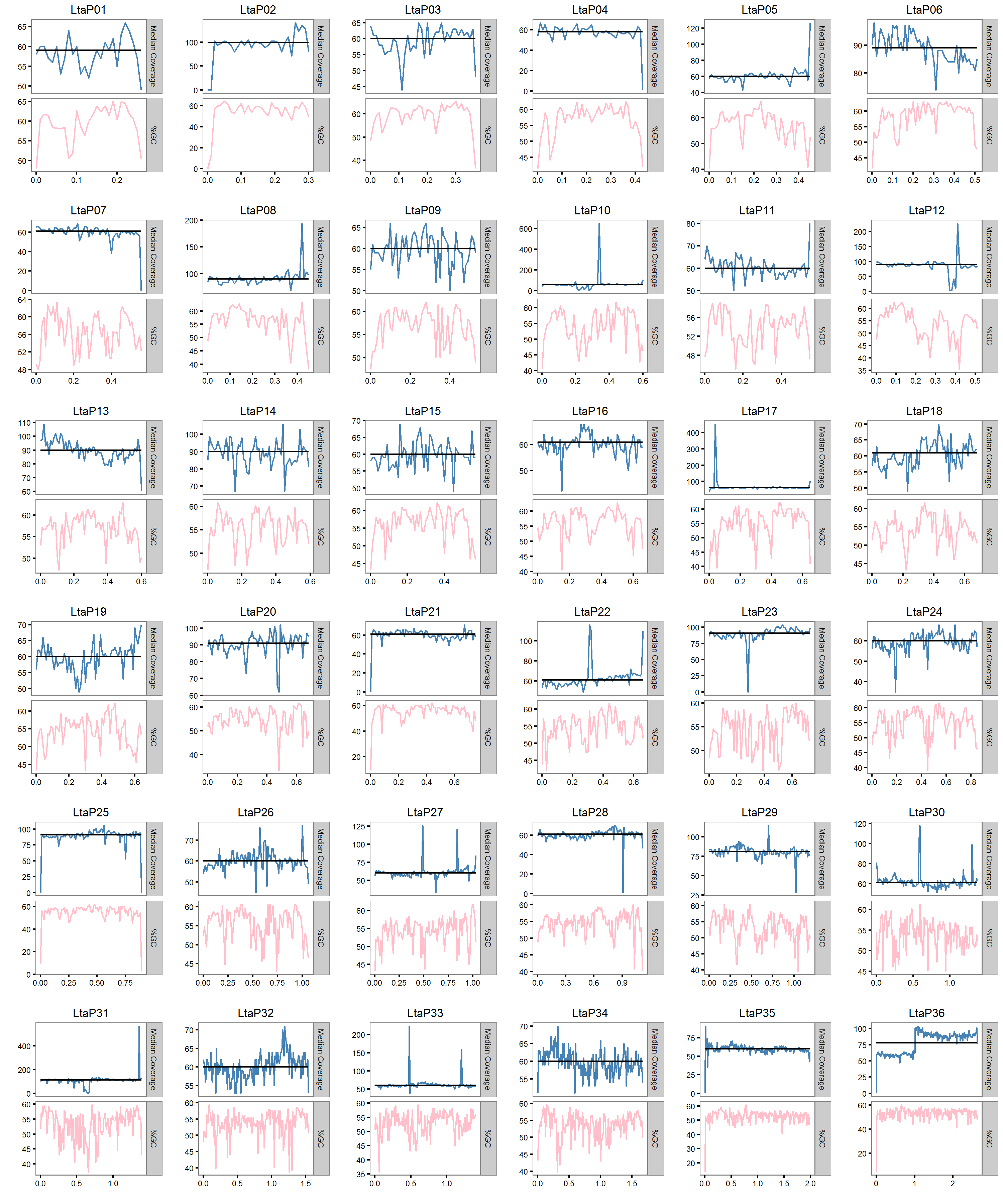


Fig. S4: Median coverage of *L. adleri* HO174 mapped to *L. tarentolae* measured in 10 Kb intervals (blue lines) for each chromosome. Black lines indicate median chromosomal coverage for that chromosome and pink lines show GC content in 10 Kb intervals.


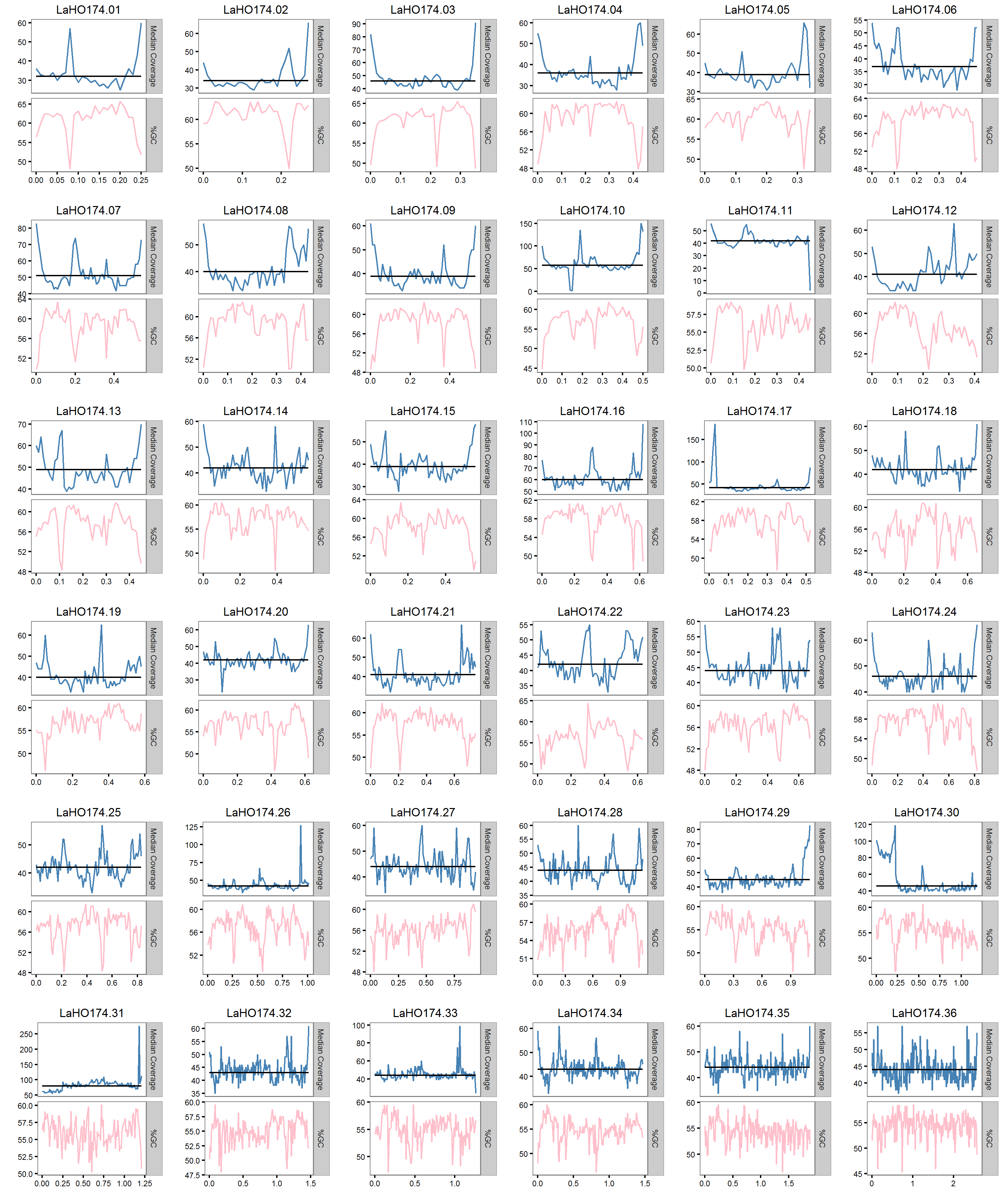


Fig. S5: Median coverage of *L. adleri* SKINK-7 mapped to *L. adleri* HO174 with unbroken chromosomes 30 and 36 measured in 10 Kb intervals (blue lines) for each chromosome. Black lines indicate median chromosomal coverage for that chromosome and pink lines show GC content in 10 Kb intervals.


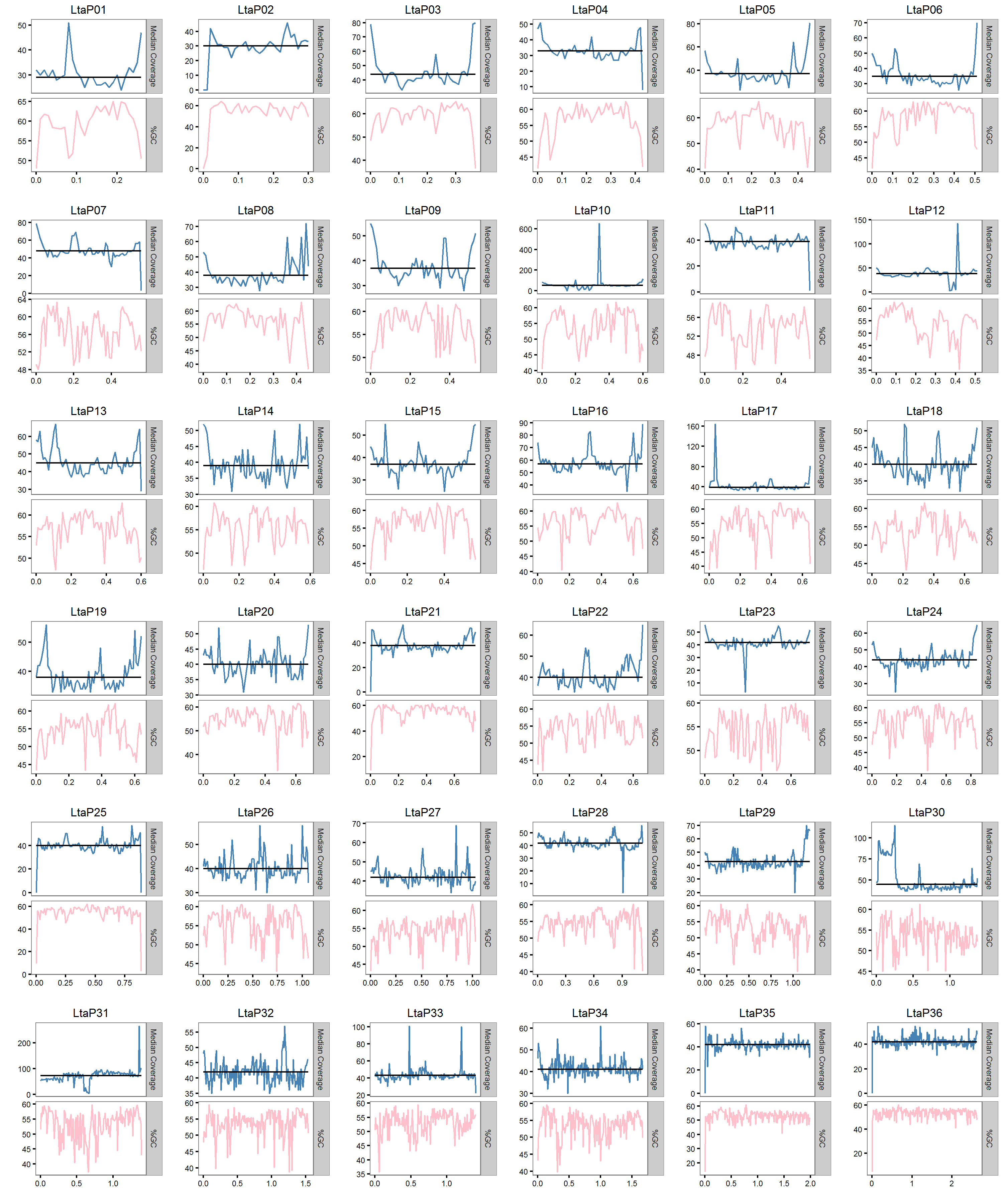


Fig. S6: Median coverage of *L. adleri* SKINK-7 mapped to *L. tarentolae* measured in 10 Kb intervals (blue lines) for each chromosome. Black lines indicate median chromosomal coverage for that chromosome and pink lines show GC content in 10 Kb intervals.


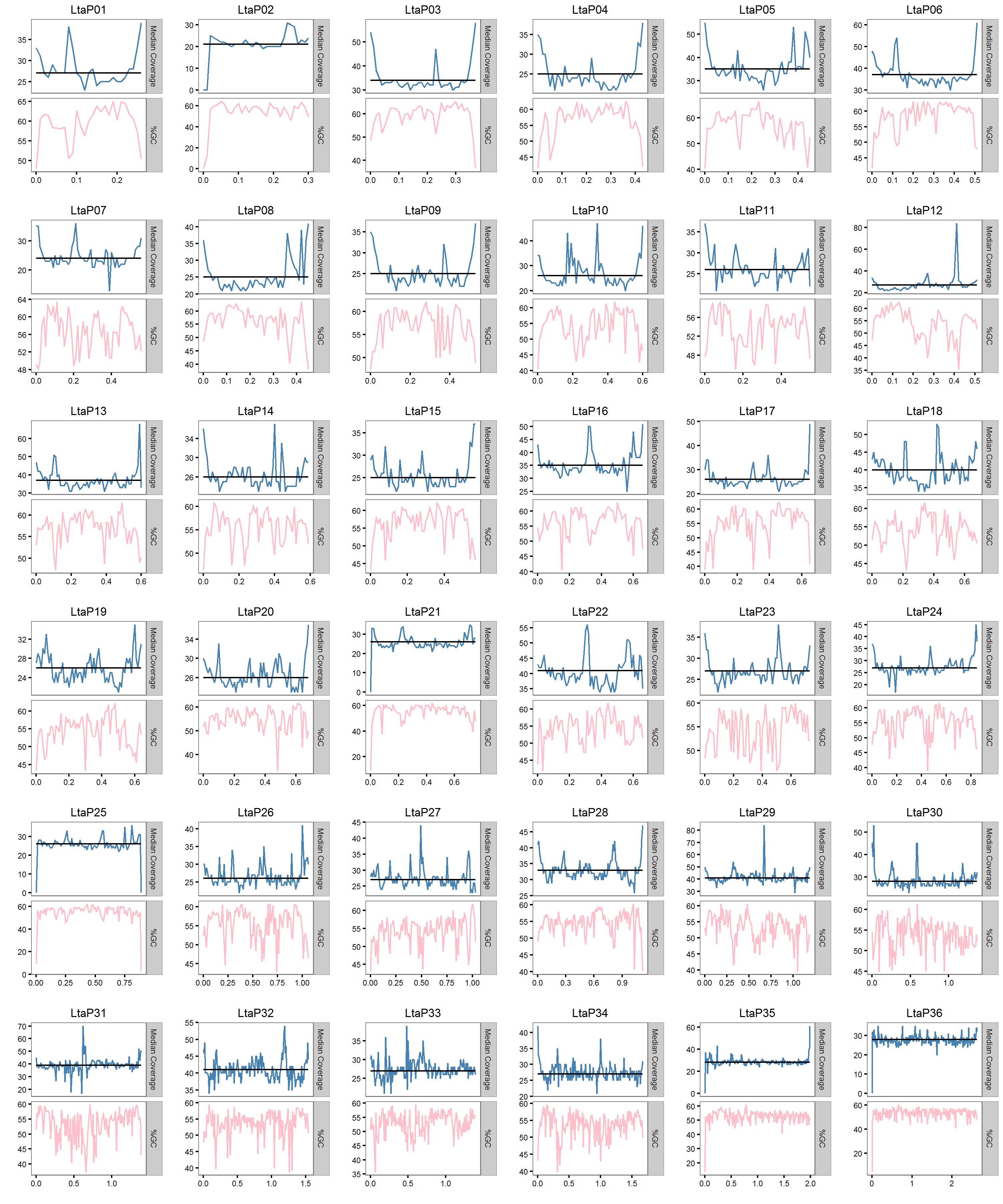


Fig. S7: Median coverage of *L. tarentolae* mapped to itself measured in 10 Kb intervals (blue lines) for each chromosome. Black lines indicate median chromosomal coverage for that chromosome and pink lines show GC content in 10 Kb intervals.


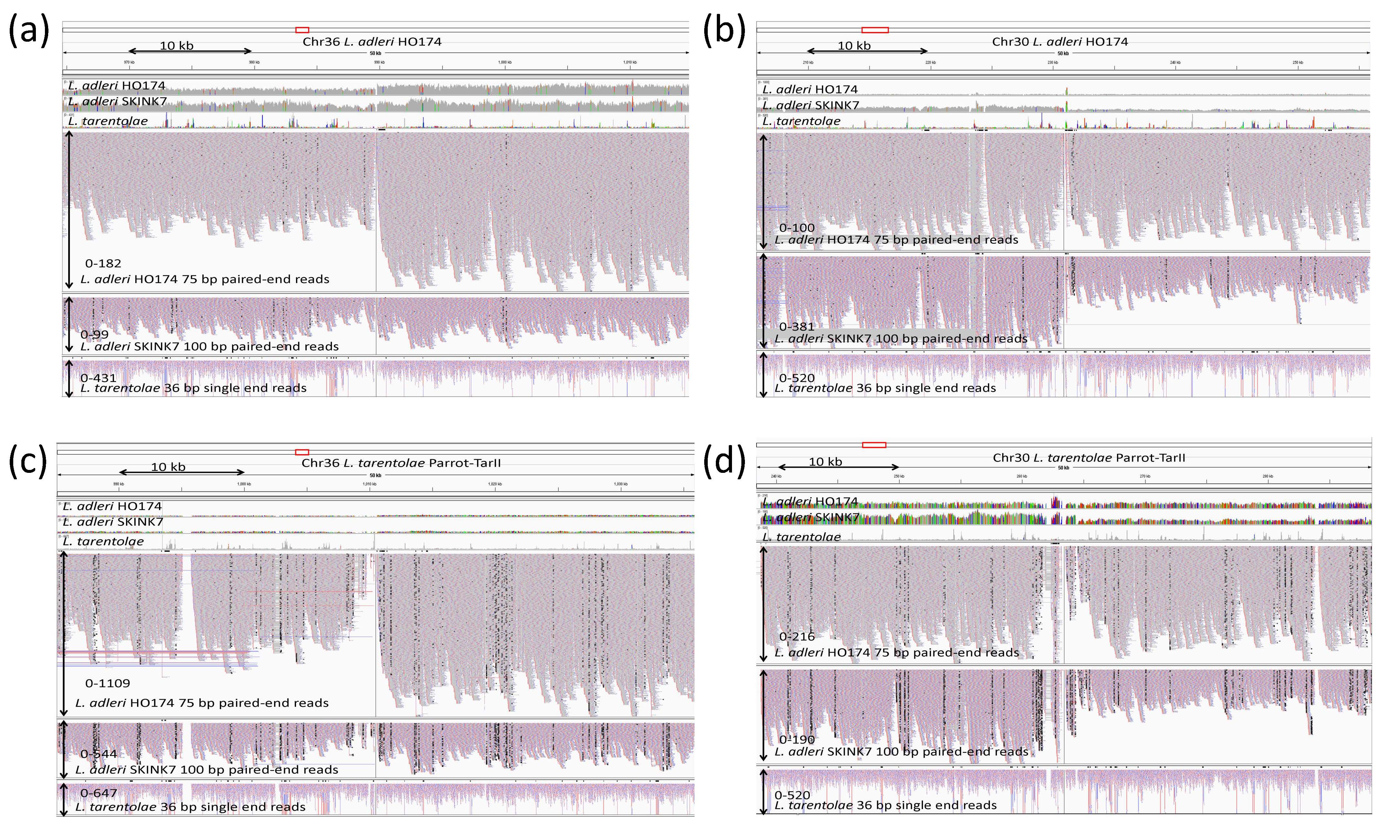


Fig. S8: Screenshot of the IGV browser showing chromosomes 30 and 36 over 50 Kb windows with *L. adleri* HO174 (first), *L. adleri* SKINK-7 (second) and *L. tarentolae* Parrot-TarII (third) reads mapped to reference sequences for the: (a) HO174 chromosome 36 with a gap at bases 989,697-989,797; (b) HO174 chromosome 30 with a gap at bases 230,911-231,011; (c) Parrot-TarII chromosome 36 with a 150 bp gap at bases 1,010,425-1,010,575; and (d) Parrot-TarII chromosome 30 with a 150 bp gap at bases 264,379-264,529. The regions shown are homologous, including the gaps on chromosomes 30 and 36 in HO174 and Parrot-TarII. The coverage of each set of reads is represented in the upper three panels of each figure and on the left-hand scale with the numbers representing the minimum and maximum coverage for the view, such as 0-182 for *L. adleri* HO174 in (a). Read colours indicate the read strand with red for positive strand reads (5’ to 3’) and blue for reverse strand (reverse complement) reads and grey lines join the mates of mapped read pairs. Vertical lines are midway through each gap and the 5’ ends of these gaps are the fission breakpoints. (a) and (c) show a coverage increase 3’ end of the chromosome 36 gap in HO174, but not for SKINK-7 or Parrot-TarII. (b) and (d) show a coverage increase 3’ end of the chromosome 30 gap in SKINK-7, but not in HO174 or Parrot-TarII.


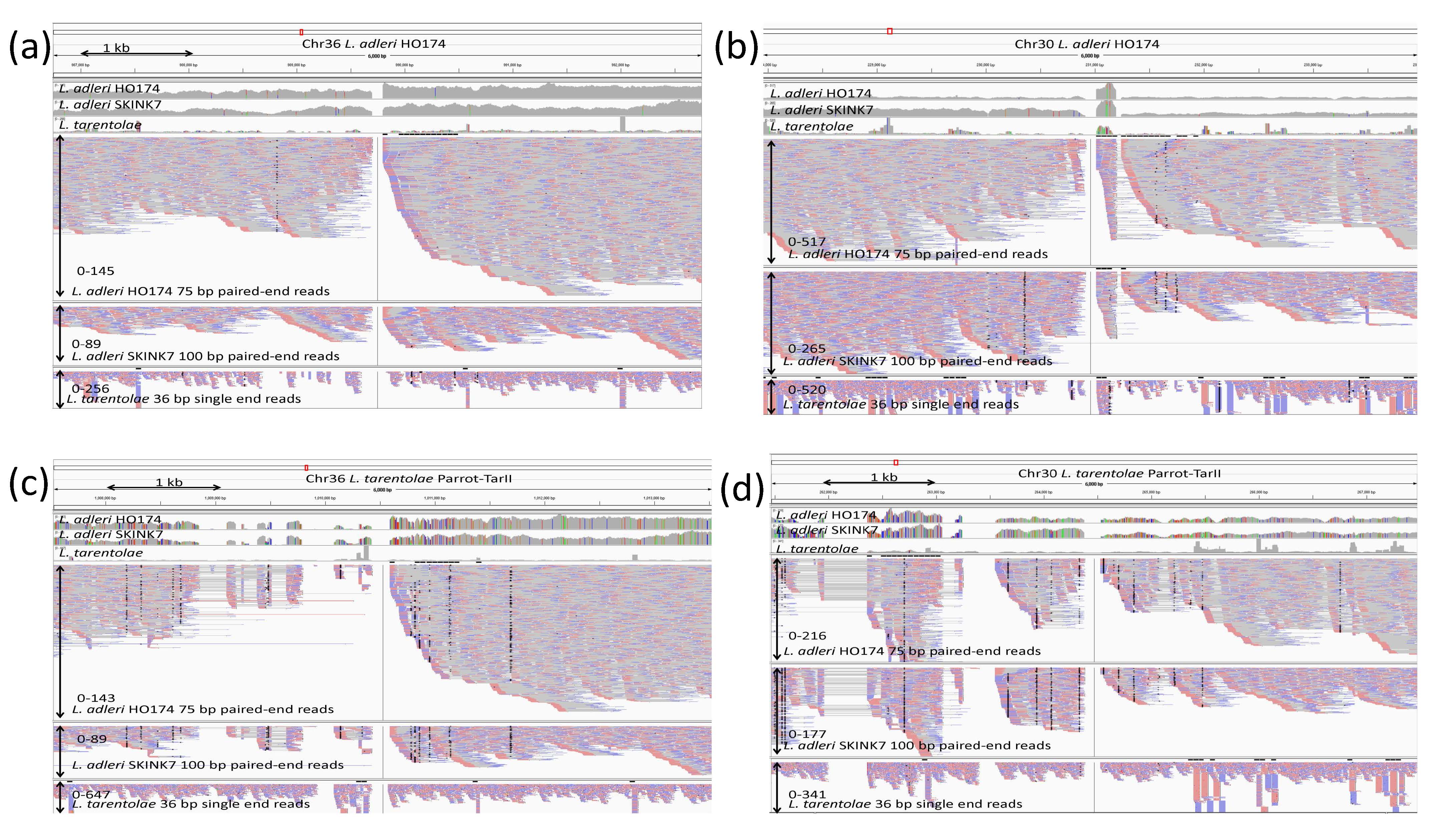


Fig. S9: Screenshot of the IGV browser showing chromosomes 30 and 36 over 6 Kb windows with *L. adleri* HO174 (first), *L. adleri* SKINK-7 (second) and *L. tarentolae* Parrot-TarII (third) reads mapped to reference sequences for the: (a) HO174 chromosome 36 with a gap at bases 989,697-989,797; (b) HO174 chromosome 30 with a gap at bases 230,911-231,011; (c) Parrot-TarII chromosome 36 with a 150 bp gap at bases 1,010,425-1,010,575; and (d) Parrot-TarII chromosome 30 with a 150 bp gap at bases 264,379-264,529. The regions shown are homologous, including the gaps on chromosomes 30 and 36 in HO174 and Parrot-TarII. The coverage of each set of reads is represented in the upper three panels of each figure and on the left-hand scale with the numbers representing the minimum and maximum coverage for the view, such as 0-145 for *L. adleri* HO174 in (a). Read colours indicate the read strand with red for positive strand reads (5’ to 3’) and blue for reverse strand (reverse complement) reads and grey lines join the mates of mapped read pairs. Vertical lines are midway through each gap and the 5’ ends of these gaps are the fission breakpoints. (a) and (c) show a coverage increase 3’ end of the chromosome 36 gap in HO174, but not for SKINK-7 or Parrot-TarII. (b) and (d) show a coverage increase 3’ end of the chromosome 30 gap in SKINK-7, but not in HO174 or Parrot-TarII. *L. adleri* HO174 and SKINK-7 paired end reads have their mates joined by grey lines to show the location of both mapped mates of a pair: this shows no read pairs cross the fission breakpoints in HO174 (a) or Parrot-TarII chromosome 36 (c). For Parrot-TarII chromosome 30, no read pairs cross the fission breakpoint but for HO174 chromosome 30, one read pair crosses the breakpoint in the SKINK-7 reads. However, this read pair has an insert size of 57,782 bp indicating that it is not properly paired.


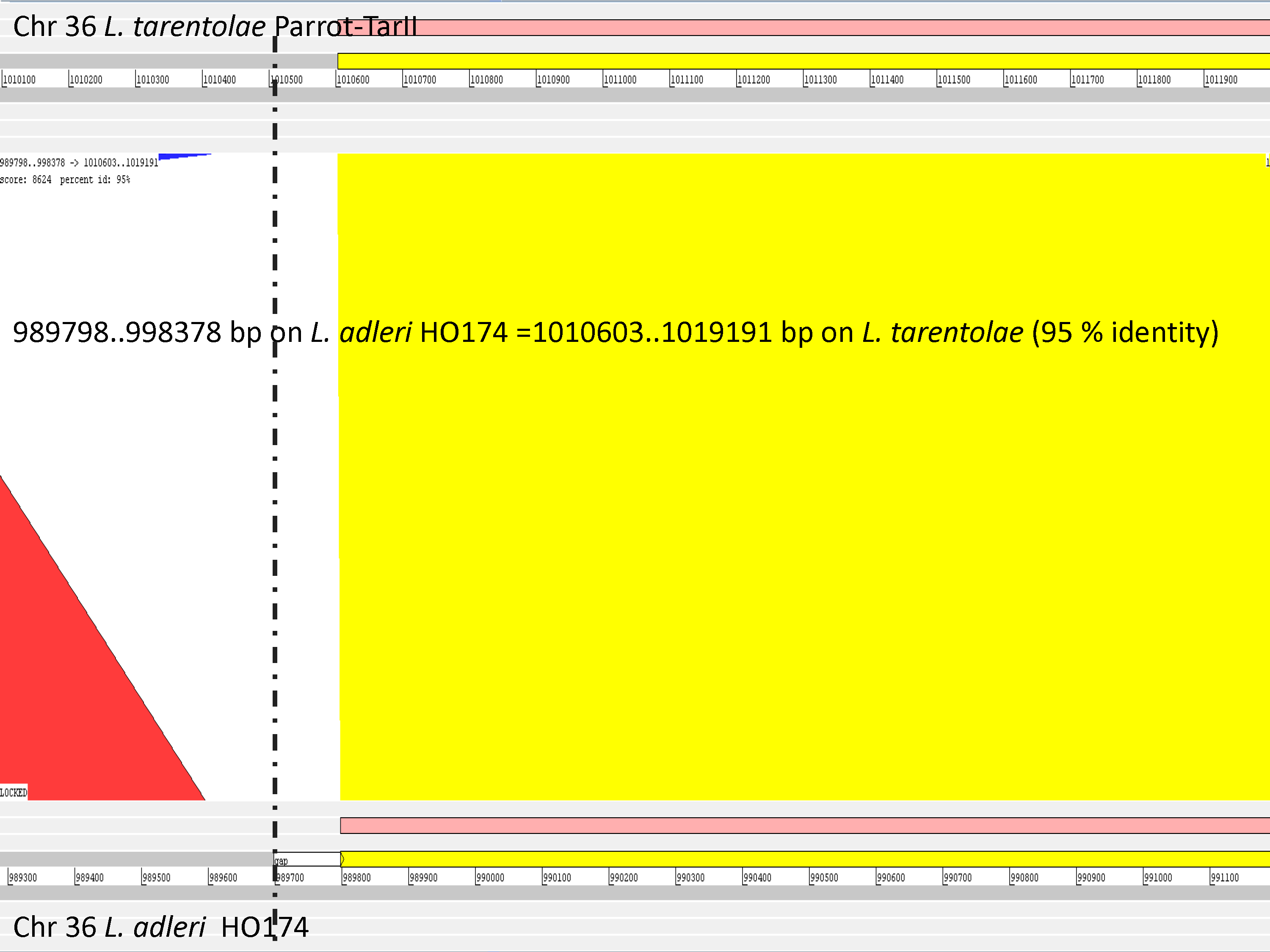


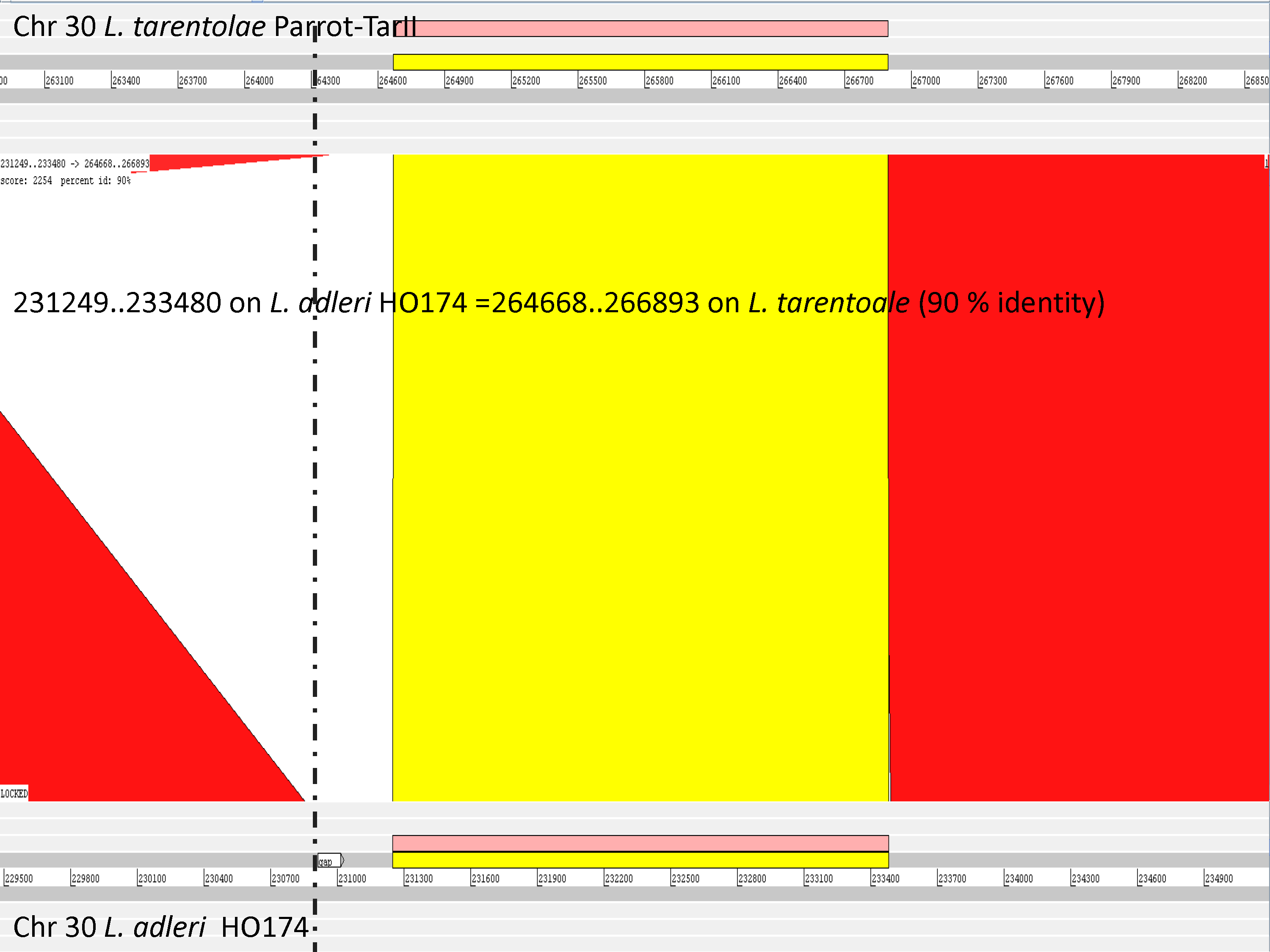


Fig. S10: Artemis Comparison Tool screenshots of homologous segments of *L. tarentolae* Parrot-TarII chromosomes 30 (top) and 36 (bottom) aligned with *L. adleri* HO174. The dashed black lines indicate the fission breakpoints. The yellow segment in each plot indicates the nearest homologous *L. tarentolae* segments.


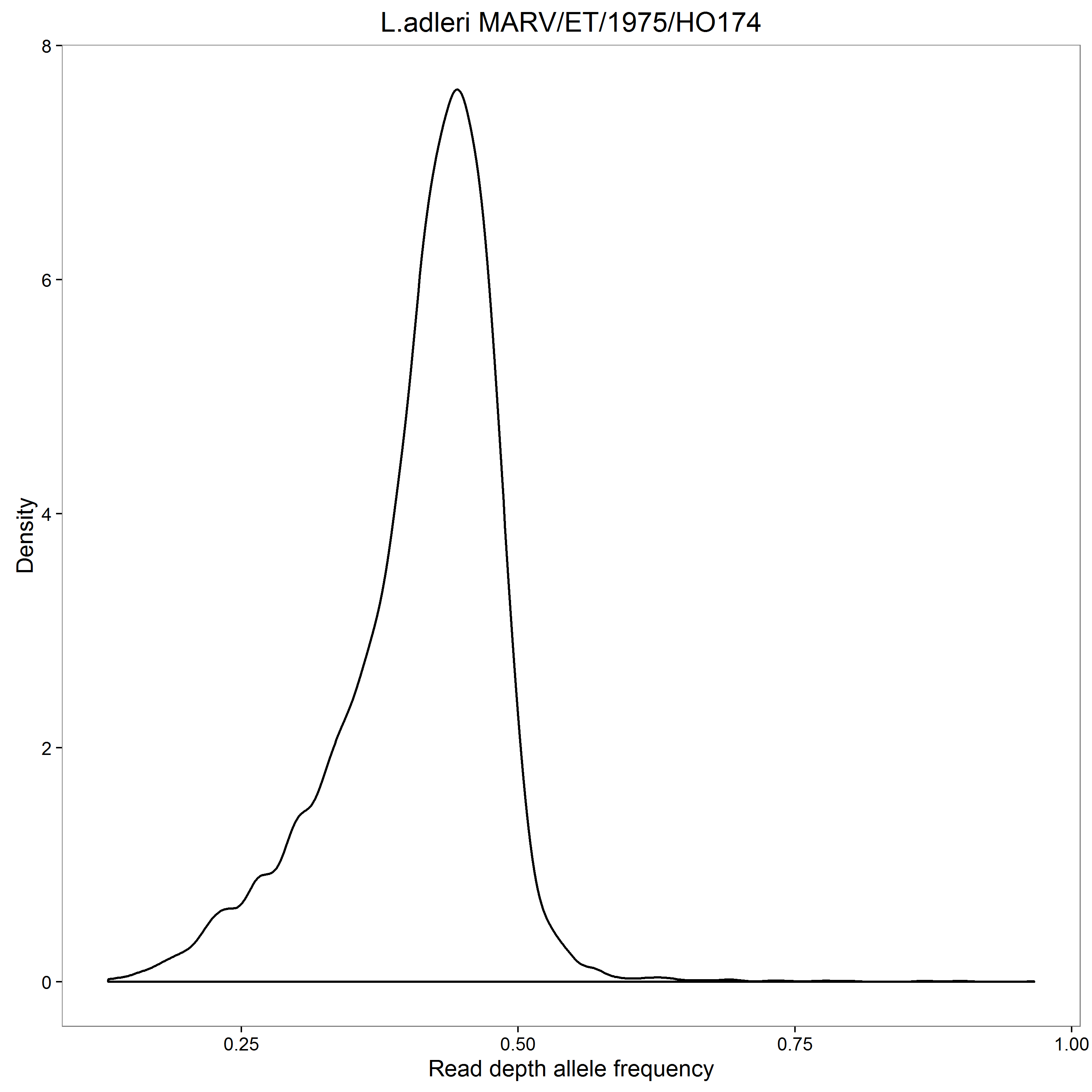


Fig. S11: Density plot of read depth allele frequency distribution of heterozygous SNPs called from self-mapped reads for *L. adleri* HO174.


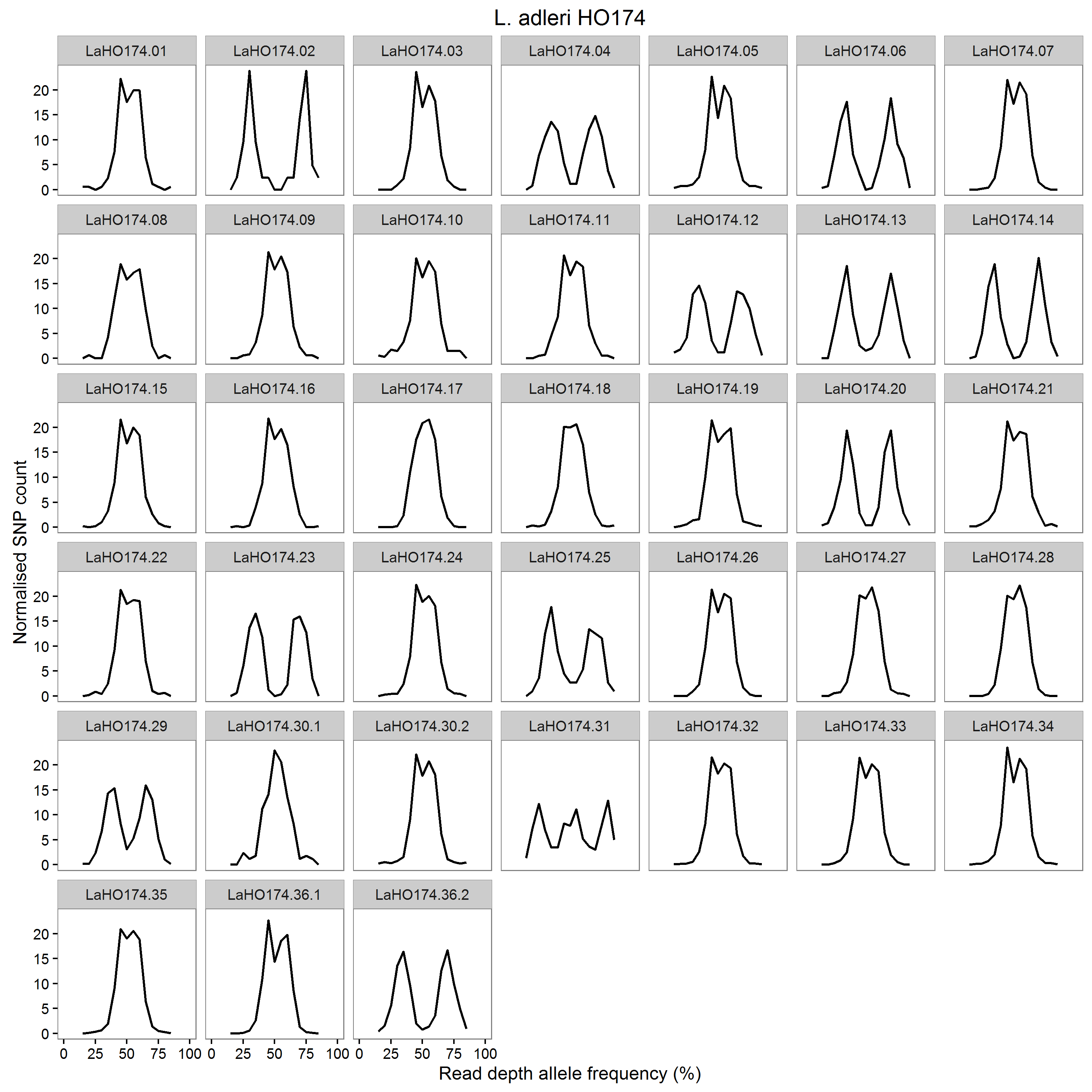


Fig. S12: Read depth allele frequency distributions for each chromosome of *L. adleri* HO174 based on heterozygous SNPs called from self mapped reads


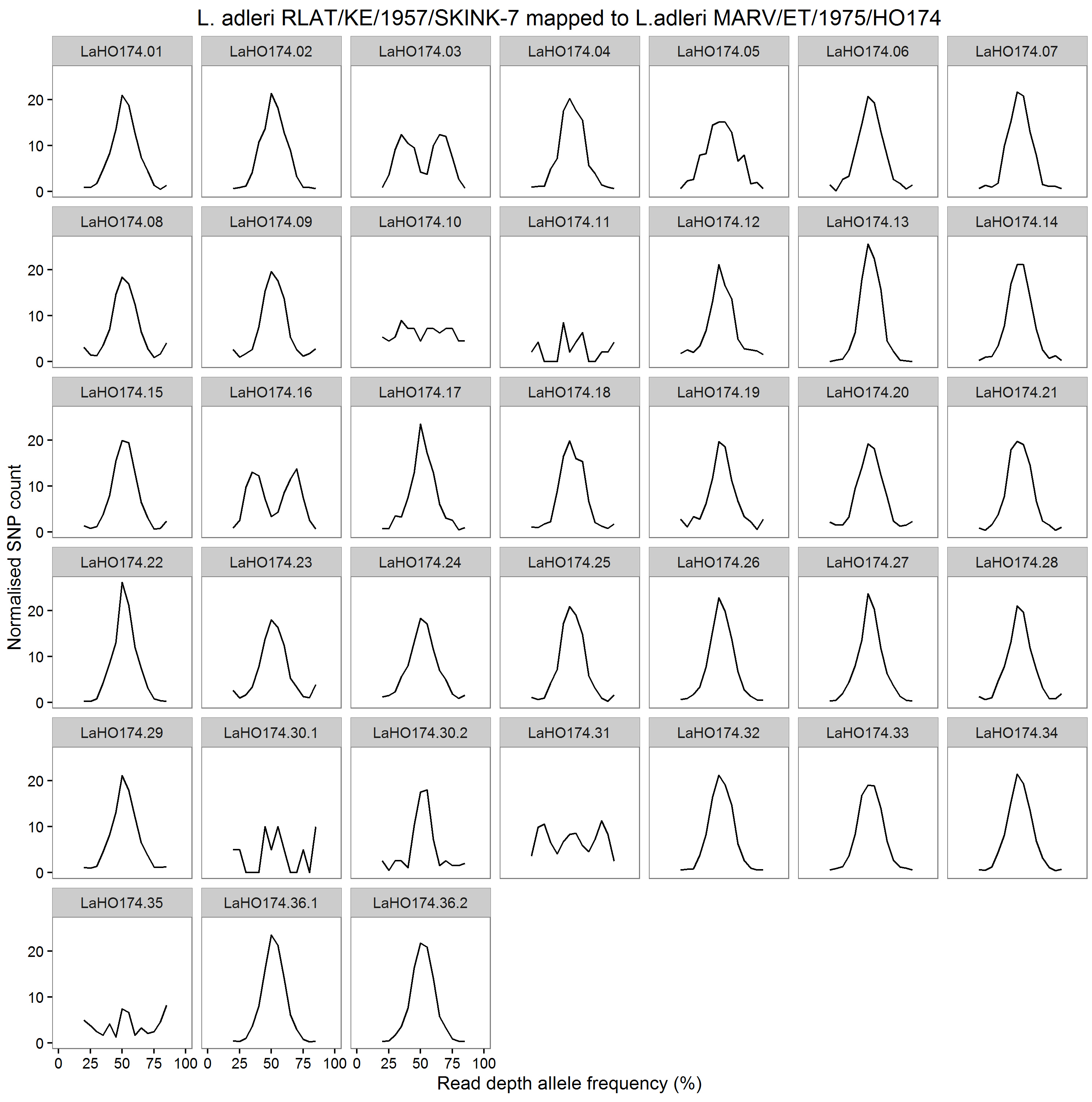


Fig. S13: Read depth allele frequency distributions for each chromosome *of L. adleri* SKINK-7 based on heterozygous SNPs called from its reads mapped to *L. adleri* HO174


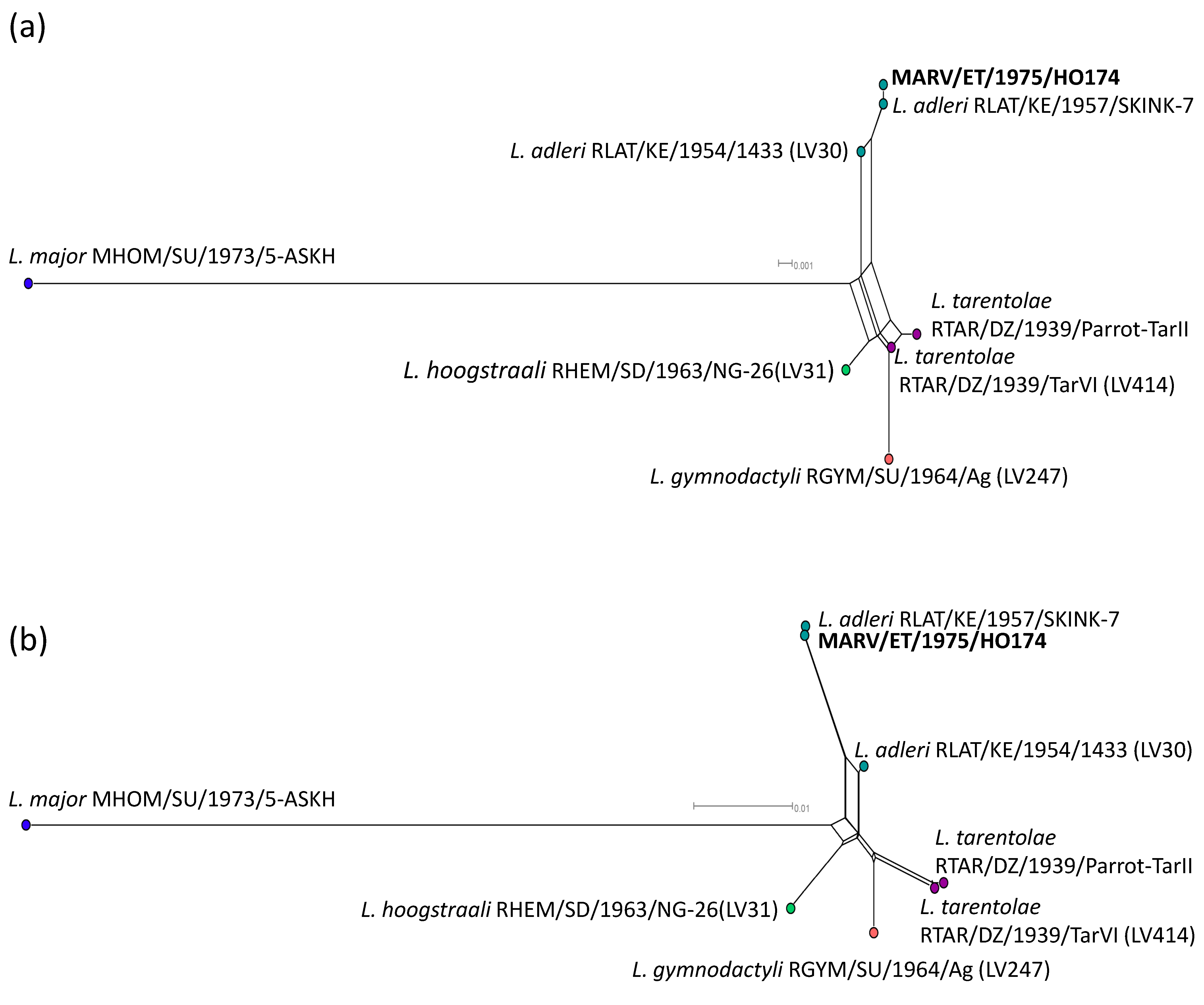


Fig. S14. (a) NeighborNet network based on uncorrected p-distances of the DNA polymerase *α* catalytic polypeptide (924 sites in alignment) gene sequences from Croan et al., 1997 with *L. major* as an outgroup. The scale bar indicates the number of substitutions per site. There is 1 substitution between HO174 and *L. adleri* SKINK-7, 4 substitutions between *L. adleri* 1433 and HO174, 15 between HO174 and each of the two *L. tarentolae* species, 17 between *L. hoogstraali* NG-26 and HO174, 21 between *L. gymnodactyli* Ag, 11 between *L. adleri* 1433 (LV30) and *L. tarentolae* TarVI and 73 between *L. major* 5-ASKH and HO174.

(b) NeighborNet network based on uncorrected p-distances of the RNA polymerase II largest subunit (1268 sites in alignment) gene sequences from Croan et al., 1997 with *L. major* as an outgroup. There is 1 substitution between HO174 and *L. adleri* SKINK-7, 17 substitutions between *L. adleri* 1433 and HO174, 34 each between *L. tarentolae* TarVI, *L. tarentolae* ParrotTar-II*, L. hoogstraali* NG-26 and *L. gymnodactyli* Ag compared with HO174, 19 between *L. adleri* 1433 and *L. tarentolae* TarVI and 130 between *L. major* 5-ASKH and HO174.

**Supplementary References:**

Aebischer, T., C. L. Bennett, M. Pelizzola, C. Vizzardelli, N. Pavelka, M. Urbano, M. Capozzoli, A. Luchini, T. Ilg, F. Granucci, C. C. Blackburn, and P. Ricciardi-Castagnoli. 2005. 'A critical role for lipophosphoglycan in proinflammatory responses of dendritic cells to Leishmania mexicana', *Eur J Immunol*, 35: 476-86.

Almeida, R., B. J. Gilmartin, S. H. McCann, A. Norrish, A. C. Ivens, D. Lawson, M. P. Levick, D. F. Smith, S. D. Dyall, D. Vetrie, T. C. Freeman, R. M. Coulson, I. Sampaio, H. Schneider, and J. M. Blackwell. 2004. 'Expression profiling of the Leishmania life cycle: cDNA arrays identify developmentally regulated genes present but not annotated in the genome', *Mol Biochem Parasitol*, 136: 87-100.

Araujo-Santos, J. M., A. Parodi-Talice, S. Castanys, and F. Gamarro. 2005. 'The overexpression of an intracellular ABCA-like transporter alters phospholipid trafficking in Leishmania', *Biochem Biophys Res Commun*, 330: 349-55.

Ashutosh, S. Sundar, and N. Goyal. 2007. 'Molecular mechanisms of antimony resistance in Leishmania', *J Med Microbiol*, 56: 143-53.

Becker, I., N. Salaiza, M. Aguirre, J. Delgado, N. Carrillo-Carrasco, L. G. Kobeh, A. Ruiz, R. Cervantes, A. P. Torres, N. Cabrera, A. Gonzalez, C. Maldonado, and A. Isibasi. 2003. 'Leishmania lipophosphoglycan (LPG) activates NK cells through toll-like receptor-2', *Mol Biochem Parasitol*, 130: 65-74.

Bessat, M., G. Knudsen, A. L. Burlingame, and C. C. Wang. 2013. 'A minimal anaphase promoting complex/cyclosome (APC/C) in Trypanosoma brucei', *PLoS One*, 8: e59258.

Brochu, C., A. Haimeur, and M. Ouellette. 2004. 'The heat shock protein HSP70 and heat shock cognate protein HSC70 contribute to antimony tolerance in the protozoan parasite leishmania', *Cell Stress Chaperones*, 9: 294-303.

Brotherton, M. C., S. Bourassa, P. Leprohon, D. Legare, G. G. Poirier, A. Droit, and M. Ouellette. 2013. 'Proteomic and genomic analyses of antimony resistant Leishmania infantum mutant', *PLoS One*, 8: e81899.

Buxbaum, L. U., H. Denise, G. H. Coombs, J. Alexander, J. C. Mottram, and P. Scott. 2003. 'Cysteine protease B of Leishmania mexicana inhibits host Th1 responses and protective immunity', *J Immunol*, 171: 3711-7.

Casgrain, P. A., C. Martel, W. R. McMaster, J. C. Mottram, M. Olivier, and A. Descoteaux. 2016. 'Cysteine Peptidase B Regulates Leishmania mexicana Virulence through the Modulation of GP63 Expression', *PLoS Pathog*, 12: e1005658.

Chowdhury, S., R. Mukhopadhyay, S. Saha, A. Mishra, S. Sengupta, S. Roy, and H. K. Majumder. 2014. 'Flavone-resistant Leishmania donovani overexpresses LdMRP2 transporter in the parasite and activates host MRP2 on macrophages to circumvent the flavone-mediated cell death', *J Biol Chem*, 289: 16129-47.

d'Avila-Levy, C. M., F. A. Marinho, L. O. Santos, J. L. Martins, A. L. Santos, and M. H. Branquinha. 2006. 'Antileishmanial activity of MDL 28170, a potent calpain inhibitor', *Int J Antimicrob Agents*, 28: 138-42.

Debrabant, A., N. Lee, G. P. Pogue, D. M. Dwyer, and H. L. Nakhasi. 2002. 'Expression of calreticulin P-domain results in impairment of secretory pathway in Leishmania donovani and reduced parasite survival in macrophages', *Int J Parasitol*, 32: 1423-34.

Dillon, L. A., K. Okrah, V. K. Hughitt, R. Suresh, Y. Li, M. C. Fernandes, A. T. Belew, H. Corrada Bravo, D. M. Mosser, and N. M. El-Sayed. 2015. 'Transcriptomic profiling of gene expression and RNA processing during Leishmania major differentiation', *Nucleic Acids Res*, 43: 6799-813.

El Baidouri, F., L. Diancourt, V. Berry, F. Chevenet, F. Pratlong, P. Marty, and C. Ravel. 2013. 'Genetic structure and evolution of the Leishmania genus in Africa and Eurasia: what does MLSA tell us', *PLoS Negl Trop Dis*, 7: e2255.

Eme, L., Trilles, A., Moreira, D., Brochier-Armanet, C. 2011. 'The phylogenomic analysis of the anaphase promoting complex and its targets points to complex and modern-like control of the cell cycle in the last common ancestor of eukaryotes', *BMC Evol Biol*, 11: 265.

Gamarro, F., M. J. Chiquero, M. V. Amador, D. Legare, M. Ouellette, and S. Castanys. 1994. 'P-glycoprotein overexpression in methotrexate-resistant Leishmania tropica', *Biochem Pharmacol*, 47: 1939-47.

Haimeur, A., C. Brochu, P. Genest, B. Papadopoulou, and M. Ouellette. 2000. 'Amplification of the ABC transporter gene PGPA and increased trypanothione levels in potassium antimonyl tartrate (SbIII) resistant Leishmania tarentolae', *Mol Biochem Parasitol*, 108: 131-5.

Holzer, T. R., W. R. McMaster, and J. D. Forney. 2006. 'Expression profiling by whole-genome interspecies microarray hybridization reveals differential gene expression in procyclic promastigotes, lesion-derived amastigotes, and axenic amastigotes in Leishmania mexicana', *Mol Biochem Parasitol*, 146: 198-218.

Ivens, A. C., C. S. Peacock, E. A. Worthey, L. Murphy, G. Aggarwal, M. Berriman, E. Sisk, M. A. Rajandream, E. Adlem, R. Aert, A. Anupama, Z. Apostolou, P. Attipoe, N. Bason, C. Bauser, A. Beck, S. M. Beverley, G. Bianchettin, K. Borzym, G. Bothe, C. V. Bruschi, M. Collins, E. Cadag, L. Ciarloni, C. Clayton, R. M. Coulson, A. Cronin, A. K. Cruz, R. M. Davies, J. De Gaudenzi, D. E. Dobson, A. Duesterhoeft, G. Fazelina, N. Fosker, A. C. Frasch, A. Fraser, M. Fuchs, C. Gabel, A. Goble, A. Goffeau, D. Harris, C. Hertz-Fowler, H. Hilbert, D. Horn, Y. Huang, S. Klages, A. Knights, M. Kube, N. Larke, L. Litvin, A. Lord, T. Louie, M. Marra, D. Masuy, K. Matthews, S. Michaeli, J. C. Mottram, S. Muller-Auer, H. Munden, S. Nelson, H. Norbertczak, K. Oliver, S. O'Neil, M. Pentony, T. M. Pohl, C. Price, B. Purnelle, M. A. Quail, E. Rabbinowitsch, R. Reinhardt, M. Rieger, J. Rinta, J. Robben, L. Robertson, J. C. Ruiz, S. Rutter, D. Saunders, M. Schafer, J. Schein, D. C. Schwartz, K. Seeger, A. Seyler, S. Sharp, H. Shin, D. Sivam, R. Squares, S. Squares, V. Tosato, C. Vogt, G. Volckaert, R. Wambutt, T. Warren, H. Wedler, J. Woodward, S. Zhou, W. Zimmermann, D. F. Smith, J. M. Blackwell, K. D. Stuart, B. Barrell, and P. J. Myler. 2005. 'The genome of the kinetoplastid parasite, Leishmania major', *Science*, 309: 436-42.

Katakura, K., H. Fujise, K. Takeda, O. Kaneko, M. Torii, M. Suzuki, K. P. Chang, and Y. Hashiguchi. 2004. 'Overexpression of LaMDR2, a novel multidrug resistance ATP-binding cassette transporter, causes 5-fluorouracil resistance in Leishmania amazonensis', *FEBS Lett*, 561: 207-12.

Kaur, J., R. Tiwari, A. Kumar, and N. Singh. 2011. 'Bioinformatic Analysis of Leishmania donovani Long-Chain Fatty Acid-CoA Ligase as a Novel Drug Target', *Mol Biol Int*, 2011: 278051.

Kedzierski, L., J. Montgomery, D. Bullen, J. Curtis, E. Gardiner, A. Jimenez-Ruiz, and E. Handman. 2004. 'A leucine-rich repeat motif of Leishmania parasite surface antigen 2 binds to macrophages through the complement receptor 3', *J Immunol*, 172: 4902-6.

Kobe, B., and A. V. Kajava. 2001. 'The leucine-rich repeat as a protein recognition motif', *Curr Opin Struct Biol*, 11: 725-32.

Kolli, B. K., J. Kostal, O. Zaborina, A. M. Chakrabarty, and K. P. Chang. 2008. 'Leishmania-released nucleoside diphosphate kinase prevents ATP-mediated cytolysis of macrophages', *Mol Biochem Parasitol*, 158: 163-75.

Leifso, K., G. Cohen-Freue, N. Dogra, A. Murray, and W. R. McMaster. 2007. 'Genomic and proteomic expression analysis of Leishmania promastigote and amastigote life stages: the Leishmania genome is constitutively expressed', *Mol Biochem Parasitol*, 152: 35-46.

Leprohon, P., D. Legare, I. Girard, B. Papadopoulou, and M. Ouellette. 2006. 'Modulation of Leishmania ABC protein gene expression through life stages and among drug-resistant parasites', *Eukaryot Cell*, 5: 1713-25.

Leprohon, P., D. Legare, and M. Ouellette. 2009. 'Intracellular localization of the ABCC proteins of Leishmania and their role in resistance to antimonials', *Antimicrob Agents Chemother*, 53: 2646-9.

Leprohon, P., D. Legare, F. Raymond, E. Madore, G. Hardiman, J. Corbeil, and M. Ouellette. 2009. 'Gene expression modulation is associated with gene amplification, supernumerary chromosomes and chromosome loss in antimony-resistant Leishmania infantum', *Nucleic Acids Res*, 37: 1387-99.

Llanes, A., C. M. Restrepo, G. Del Vecchio, F. J. Anguizola, and R. Lleonart. 2015. 'The genome of Leishmania panamensis: insights into genomics of the L. (Viannia) subgenus', *Sci Rep*, 5: 8550.

Lodge, R., and A. Descoteaux. 2008. 'Leishmania invasion and phagosome biogenesis', *Subcell Biochem*, 47: 174-81.

Mahmoudzadeh-Niknam, H., and J. H. McKerrow. 2004. 'Leishmania tropica: cysteine proteases are essential for growth and pathogenicity', *Exp Parasitol*, 106: 158-63.

Matte, C., P. A. Casgrain, O. Seguin, N. Moradin, W. J. Hong, and A. Descoteaux. 2016. 'Leishmania major Promastigotes Evade LC3-Associated Phagocytosis through the Action of GP63', *PLoS Pathog*, 12: e1005690.

McNicoll, F., J. Drummelsmith, M. Muller, E. Madore, N. Boilard, M. Ouellette, and B. Papadopoulou. 2006. 'A combined proteomic and transcriptomic approach to the study of stage differentiation in Leishmania infantum', *Proteomics*, 6: 3567-81.

Mottram, J. C., G. H. Coombs, and J. Alexander. 2004. 'Cysteine peptidases as virulence factors of Leishmania', *Curr Opin Microbiol*, 7: 375-81.

Nandan, D., T. Yi, M. Lopez, C. Lai, and N. E. Reiner. 2002. 'Leishmania EF-1alpha activates the Src homology 2 domain containing tyrosine phosphatase SHP-1 leading to macrophage deactivation', *J Biol Chem*, 277: 50190-7.

Ouellette, M., J. Drummelsmith, A. El-Fadili, C. Kundig, D. Richard, and G. Roy. 2002. 'Pterin transport and metabolism in Leishmania and related trypanosomatid parasites', *Int J Parasitol*, 32: 385-98.

Peacock, C. S., K. Seeger, D. Harris, L. Murphy, J. C. Ruiz, M. A. Quail, N. Peters, E. Adlem, A. Tivey, M. Aslett, A. Kerhornou, A. Ivens, A. Fraser, M. A. Rajandream, T. Carver, H. Norbertczak, T. Chillingworth, Z. Hance, K. Jagels, S. Moule, D. Ormond, S. Rutter, R. Squares, S. Whitehead, E. Rabbinowitsch, C. Arrowsmith, B. White, S. Thurston, F. Bringaud, S. L. Baldauf, A. Faulconbridge, D. Jeffares, D. P. Depledge, S. O. Oyola, J. D. Hilley, L. O. Brito, L. R. Tosi, B. Barrell, A. K. Cruz, J. C. Mottram, D. F. Smith, and M. Berriman. 2007. 'Comparative genomic analysis of three Leishmania species that cause diverse human disease', *Nat Genet*, 39: 839-47.

Raymond, F., S. Boisvert, G. Roy, J. F. Ritt, D. Legare, A. Isnard, M. Stanke, M. Olivier, M. J. Tremblay, B. Papadopoulou, M. Ouellette, and J. Corbeil. 2012. 'Genome sequencing of the lizard parasite Leishmania tarentolae reveals loss of genes associated to the intracellular stage of human pathogenic species', *Nucleic Acids Res*, 40: 1131-47.

Rochette, A., F. Raymond, J. M. Ubeda, M. Smith, N. Messier, S. Boisvert, P. Rigault, J. Corbeil, M. Ouellette, and B. Papadopoulou. 2008. 'Genome-wide gene expression profiling analysis of Leishmania major and Leishmania infantum developmental stages reveals substantial differences between the two species', *BMC Genomics*, 9: 255.

Rogers, M. B., J. D. Hilley, N. J. Dickens, J. Wilkes, P. A. Bates, D. P. Depledge, D. Harris, Y. Her, P. Herzyk, H. Imamura, T. D. Otto, M. Sanders, K. Seeger, J. C. Dujardin, M. Berriman, D. F. Smith, C. Hertz-Fowler, and J. C. Mottram. 2011. 'Chromosome and gene copy number variation allow major structural change between species and strains of Leishmania', *Genome Res*, 21: 2129-42.

Shaw, C. D., J. Lonchamp, T. Downing, H. Imamura, T. M. Freeman, J. A. Cotton, M. Sanders, G. Blackburn, J. C. Dujardin, S. Rijal, B. Khanal, C. J. Illingworth, G. H. Coombs, and K. C. Carter. 2016. 'In vitro selection of miltefosine resistance in promastigotes of Leishmania donovani from Nepal: genomic and metabolomic characterization', *Mol Microbiol*, 99: 1134-48.

Valdivia, H. O., J. L. Reis-Cunha, G. F. Rodrigues-Luiz, R. P. Baptista, G. C. Baldeviano, R. V. Gerbasi, D. E. Dobson, F. Pratlong, P. Bastien, A. G. Lescano, S. M. Beverley, and D. C. Bartholomeu. 2015. 'Comparative genomic analysis of Leishmania (Viannia) peruviana and Leishmania (Viannia) braziliensis', *BMC Genomics*, 16: 715.

Van der Auwera, G., I. Maes, S. De Doncker, C. Ravel, L. Cnops, M. Van Esbroeck, A. Van Gompel, J. Clerinx, and J. C. Dujardin. 2013. 'Heat-shock protein 70 gene sequencing for Leishmania species typing in European tropical infectious disease clinics', *Euro Surveill*, 18: 20543.

Vickers, T. J., and S. M. Beverley. 2011. 'Folate metabolic pathways in Leishmania', *Essays Biochem*, 51: 63-80.

Williams, R. A., T. K. Smith, B. Cull, J. C. Mottram, and G. H. Coombs. 2012. 'ATG5 is essential for ATG8-dependent autophagy and mitochondrial homeostasis in Leishmania major', *PLoS Pathog*, 8: e1002695.

Williams, R. A., L. Tetley, J. C. Mottram, and G. H. Coombs. 2006. 'Cysteine peptidases CPA and CPB are vital for autophagy and differentiation in Leishmania mexicana', *Mol Microbiol*, 61: 655-74.

Williams, R. A., K. L. Woods, L. Juliano, J. C. Mottram, and G. H. Coombs. 2009. 'Characterization of unusual families of ATG8-like proteins and ATG12 in the protozoan parasite Leishmania major', *Autophagy*, 5: 159-72.

Yao, C., J. E. Donelson, and M. E. Wilson. 2003. 'The major surface protease (MSP or GP63) of Leishmania sp. Biosynthesis, regulation of expression, and function', *Mol Biochem Parasitol*, 132: 1-16.

Young, M. D., M. J. Wakefield, G. K. Smyth, and A. Oshlack. 2010. 'Gene ontology analysis for RNA-seq: accounting for selection bias', *Genome Biol*, 11: R14.

Zhang, W. W., and G. Matlashewski. 2012. 'Deletion of an ATP-binding cassette protein subfamily C transporter in Leishmania donovani results in increased virulence', *Mol Biochem Parasitol*, 185: 165-9.
